# Evaluation of the impact of non-slip socks on the motor recovery of elderly people in acute care hospitals: Protocol for a randomized, controlled trial study

**DOI:** 10.1371/journal.pone.0283226

**Published:** 2023-05-01

**Authors:** Thomas Rulleau, Lucie Planche, Agnès Dorion, Girolamo Soldani, Cécile Blain, Catherine Chapeleau, Yves Bleher, Cécile Da Silva, Nathalie Launeau, Emmanuelle Joguet, Ronan Fevrier, Romain Decours

**Affiliations:** 1 Unité de recherche clinique, CHD-Vendée, La Roche Sur Yon, France; 2 Service de rééducation, CHD-Vendée, La Roche Sur Yon, France; 3 Service de court séjour gériatrique, CHD-Vendée, La Roche Sur Yon, France; 4 Service de médecine post-urgence CHD-Vendée, La Roche Sur Yon, France; Prince Sattam Bin Abdulaziz University, College of Applied Medical Sciences, SAUDI ARABIA

## Abstract

**Background:**

Older patients often arrive in acute care wards with inappropriate footwear. Hospitals may provide non-slip socks to improve the patients’ safety. However, few studies have been conducted on the benefits of non-slip socks. A recent literature review found only two randomized controlled studies that evaluated non-slip socks, but the socks were not the primary focus of the studies. The aim of this study is therefore to specifically evaluate the benefits of non-slip socks on gait in hospitalized older people.

**Methods:**

This open, randomized, controlled trial will include patients aged 75 years and over, hospitalized in an acute medical unit. Patients will be randomized to either remain barefoot or wear non-slip socks throughout their stay. The primary outcome is gait speed, assessed on Day 1 and Day 8.

**Discussion:**

This randomized controlled trial should provide clinicians with a scientific rational for the recommendation, or not, of the use of non-slip socks for older patients in acute care hospitals.

**Trial registration:**

https://clinicaltrials.gov/ on May 12, 2021 under the reference: NCT04882696 https://clinicaltrials.gov/ct2/show/NCT04882696

## Introduction

Patients who are hospitalized following an unplanned admission often do not have appropriate personal belongings for their stay. In older adults, a lack of appropriate footwear can lead to hinder the rehabilitation process. Older patients in acute wards are frequently confused and fatigued, and require early rehabilitation both to facilitate their orientation [1–3] as well as to prevent falls [4]. Adequate footwear is thus essential so they can walk on the ward as soon as possible, with or without assistance [5]. A simple, low-cost solution sometimes used by hospitals is non-slip socks. However, a recent narrative review did not recommend non-slip socks due to a lack of evidence supporting their use to prevent falls, and reports that they could be a source of bacterial transmission [6]. This conclusion is, however, limited by the non-systematic methodology which meant that not all available studies of non-slip socks were included, and the fact the studies included were heterogenous, ranging from laboratory studies of young, healthy adults to older adults in hospital settings. In addition, studies of the risk of bacterial transmission have reported the presence of bacteria on socks, but did not compare this with the presence of bacteria on other types of footwear [7]. We recently conducted a systematic review of the use of non-slip socks in hospitals specifically in senior subjects [8]. We found only seven non-controlled studies and two randomized controlled trials (RCTs). The RCTs evaluated the different factors that influence falls, including non-slip socks, but the socks were not the focus of the studies. Therefore, to date, knowledge of the benefits of non-slip socks is limited. We concluded that more RCTs were required to specifically evaluate the benefits of non-slip socks as part of the management of patients on geriatric wards [8].

Firstly, it seems important to evaluate the effect of wearing non-slip socks on a patient’s functional mobility. Indeed, wearing adapted footwear can reduce the fear of falling, which breaks the vicious circle of falling—fear of falling—loss of independence [8–10]. This confidence in one’s balance should have a positive influence on functional mobility at one week.

According to simulation theory, human movement can be separated into 2 phases, a planification phase (anticipation) and an execution phase [11]. The concordance between these phases can be reliably evaluated using a simple test that evaluates the temporal concordance between imagined practice and actual physical practice [12]. This concordance is translated into an Index of Isochrony and provides an indication of motor prediction ability [13]. The degree of isochrony has been shown to be correlated with the decrease in walking speed during the dual task [14] therefore this test could indicate a risk of falls due to poor anticipation of the person’s own motor capacities. As Personnier et al. (2008) highlighted the influence of a change in task conditions on motor planning for upper limb [15], it could be interesting to evaluate task condition’s influence for lower limb. We would like to evaluate whether wearing socks could have an influence on the motor planning of patients.

Gait speed during duel task walking provides an indication of the cognitive reserve available [16]. Stopping walking or a decrease in walking speed of more than 30% in dual task conditions is predictive of falls [17]. We want to evaluate whether a week spent wearing anti-slip socks has a positive impact on this important clinical aspect of elderly patients.

Falling is an important element in the care of geriatric patients. It is defined as “an unexpected event in which the participant comes to rest on the ground, floor, or lower level” [18].

Finally, the positive impact of motor recovery could accelerate the patient’s discharge from the acute care unit. It therefore seems necessary to verify the impact of non-slip socks on the length of stay.

The primary aim of this study is therefore to compare differences in gait speed at day 8 in the controls (barefoot) to those who were managed by wearing non-slip socks while controlling for baseline gait speed. Gait speed is a recognized clinical tool to assess physical weakness and autonomy [19–21]. The secondary aims are to assess the immediate impact, and the impact after one week, of non-slip socks on short-term motor skills, short-term motor planning, dual task walking, fear of falls, occurrence of falls and length of stay.

We hypothesize that patients who wear non-slip socks will have a higher gait speed at 8 days.

## Methods

### Design

**Fig 1:**
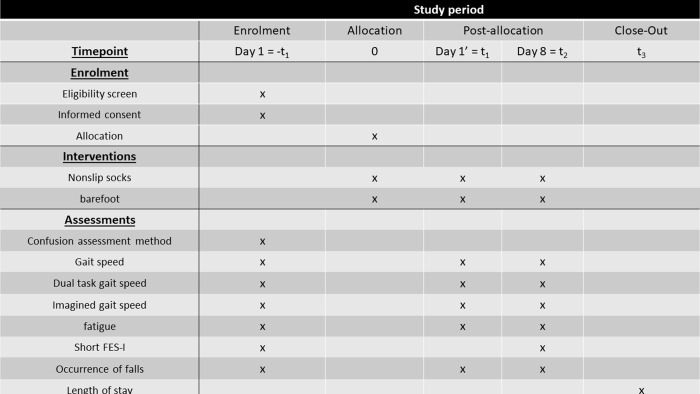
Template of content for the schedule of enrolment, interventions, and assessments.

**Fig 2:**
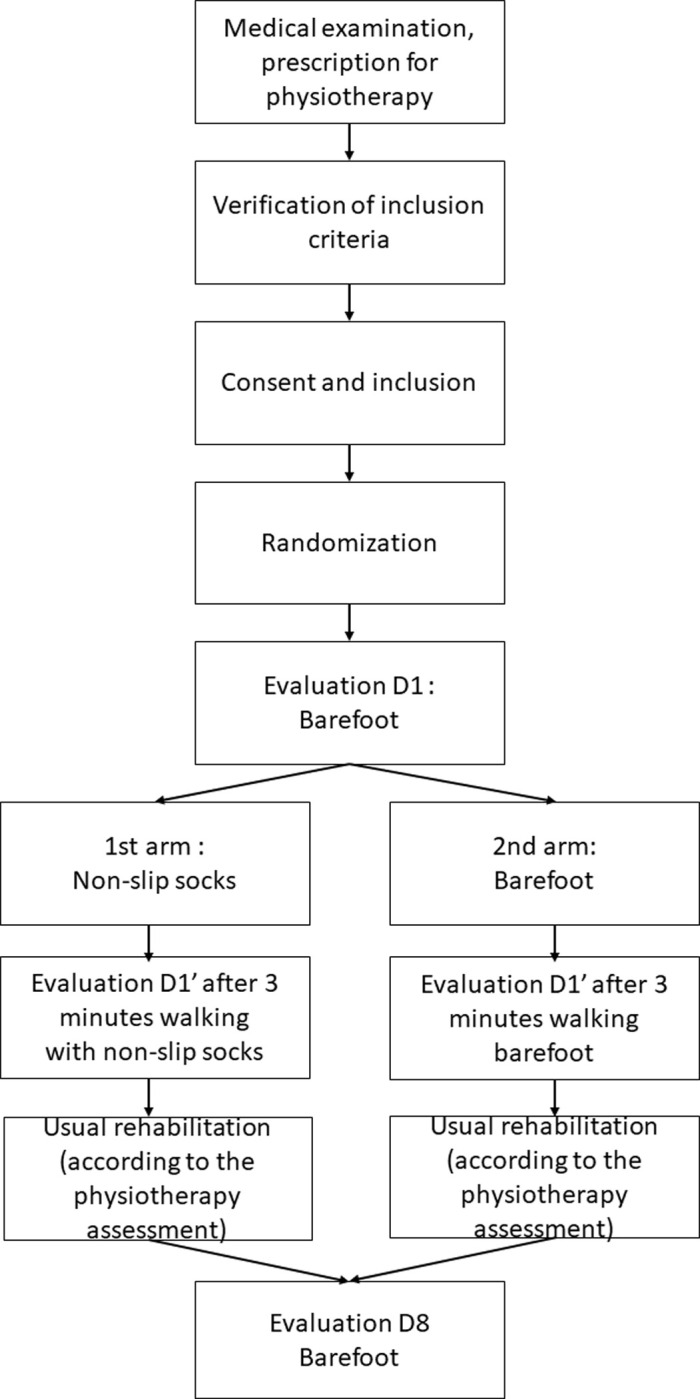
Study flow chart.

A single-center, superiority, randomized controlled open-label trial will be conducted in Vendée Departmental Hospital Center (CHD-Vendée) to compare usual barefoot care (control group) with specific management using non-slip socks (experimental group), as illustrated in Fig 1. As describe in Fig 2, barefoot care was chosen for the control arm since this corresponds to actual practice in hospitals. Socks will be provided by the hospital during and after the study. Patients have physiotherapy 3 times a week, with standard management of autonomy training, walking, balance.

The study will be coordinated by the Vendee Hospital investigation team lead by the head physician of the department. Ethical approval will be sought from our local ethics committee. This protocol has been registered on clinical trials.gov (May 12, 2021: NCT04882696, https://clinicaltrials.gov/ct2/show/NCT04882696).

### Participants

Participants will be recruited from the acute geriatric or post-emergency medicine wards of our hospital.

Patients who are considered to require physiotherapy and to have unsuitable footwear by the admitting physician will be eligible. The inclusion criteria will be checked by the study physiotherapists.

*Inclusion criteria*:

- Patients aged 75 and over,
- Patients who require physiotherapy,
- Patients with a planned length of stay of at least one week for physiotherapy.
- Patients who arrived on the ward with unsuitable footwear (assessment at the discretion of the clinician: no back support, insecure fastenings, etc.), or without footwear,
- Patients able to walk at least 10 m with or without assistive devices on admission and after physiotherapist evaluation
- Patients who have given oral consent

*Exclusion criteria*:

- Inability to understand or perform study-specific clinical tests
- Patients with a history of dementia assessed prior to hospitalization (MMSE score ≤ 22/30)
- Patients with uncorrected visual deficits
- Patient under guardianship or curatorship
- Patient participating in an interventional clinical research protocol that may affect the outcomes of this protocol.
- Patient unable to wear socks or walk barefoot (need for support stockings or socks, wound on the foot or lower leg, swollen feet, etc.)

### Sample size determination

The sample size required for this study was estimated at 46. The calculation was based on a difference of 0.10m/s in the ten-meter timed walk (see below) between the two groups, a standard deviation of the difference of 0.10 (an improvement in walking speed of 0.10m/s is considered clinically relevant in an older adults with mobility disabilities, subacute stroke survivors, and community dwelling older people [22]), an alpha risk set at 5% and a power of 90% (two sample t-test). To guarantee the power of the study, we plan to include 50 patients.

### Randomization

Patients who meet the inclusion criteria will be informed of the study. If they accept to participate and provide informed consent, a study physiotherapist will perform the randomization via a centralized server (Ennov Clinical) before performing any evaluations. A random block size randomization will be carried out in a 1:1 ratio without stratification.

### Outcomes

All assessments will be performed by physiotherapists who will also perform the patient’s rehabilitation, as illustrated in Table 1: summary of data collection by day.

**Table 1:** Summary of data collection by day.

Actions	D1	D1’ (after 3 minutes of walking)	D8	Discharge from hospital
Patient information	X			
Informed Consent Collection	X			
Verification of inclusion and non-inclusion criteria	X			
Randomization	X			
*Confusion Assessment Method*	X			
VAS fatigue	X	X	X	
Medical background (falls, MMSE etc.)	X			
Clinical examination	X		X	
Tests: • Gait speed at patient’s preferred speed over 10m (x2), • Gait speed in double task over 10m (x2), • Imagined walking speed over 10m (x2),	X	X	X	
Questionnaire Short-FESI	X		X	
Length of stay in geriatric short stay or post-emergency medicine				X

The Confusion Assessment Method scale (unless already performed as part of usual care) will be rated on D1 to characterize the patients’ confusion.

Outcomes will be evaluated on day 1 and day 8 –the same outcomes will be evaluated on both days, in the same order. Two conditions will be tested on day 1: barefoot (D1, baseline), and barefoot or with non-slip socks according to group allocation (D1’). Between the conditions, the patients will be encouraged to walk for 3 minutes either barefoot or with the socks, according to their group allocation. On D8, all patients will be evaluated barefoot.

Fatigue will be rated before and after each condition on day 1 and before and after the evaluation on day 8. Three gait assessments will be performed in each condition in the same order (approximately 10 minutes duration for each condition). Fear of falls will be evaluated after each condition.

### Confusion assessment method

This tool will be used to determine if the patient is confused. It is rated with a caregiver, and assesses 4 clinical signs suggestive of confusion [23, 24]:

1. Sudden onset and fluctuation of symptoms;
2. Inattention;
3. Disorganized thinking;
4. Impaired alertness.

If 1 and 2 and either 3 or 4 are positive, the patient is considered to be confused [24].

### Visual Analog Scale for fatigue

A Visual Analog Scale (VAS) will be used to evaluate the patient’s level of fatigue. VASs are commonly for the assessment of pain [25], and have been proposed for the evaluation of fatigue [26].

The patient will be given a VAS presented on a sliding bar and told:

"This scale represents your level of fatigue. If you put the slider all the way to the left, it will indicate that you are not tired at all. If you put it all the way to the right, it will indicate that you are incredibly tired. Please put show me with the slider how tired you are right now”. Fatigue will then be rated from 0 to 100.

### Ten-meter timed walk

The 10m timed walk will be used to evaluate spontaneous gait speed [27]. This test is commonly used in clinical practice to evaluate functional mobility. It is simple to use, even in patients with cognitive disorders or who are confused.

Floor markings will be used to provide visual cues for the start and end of the walk. The time taken to cover the 10m will be measured with a stopwatch. The test will be performed twice, and the average speed used for analysis.

Spontaneous gait speed for patients over 80 years of age is 0.943m/sec ± 0.091 [27, 28]. The best initial estimates of small meaningful changes are close to 0.05 m/s for walking speed [22] and the minimal clinically important difference is in the order of 0.10 m/s [22].

### Dual task walk

The same procedure will be used as for the 10m timed walk; however, the patient will be asked to list as many animals as possible while performing the test. The test will be performed twice, and the average speed used for analysis.

### Index of isochrony of gait speed

The test will be performed with the patient standing in front of the 10m timed walk course. They will be instructed to imagine themselves performing the timed walk test. On completion, they will say stop. The test will be performed twice, and the average speed used for analysis.

### Fear of falling

The Short-Falls Efficacy Scale International (Short-FES-I) is a self-administered, patient-completed questionnaire consisting of 7 items, each with 4 possible answers. It has excellent internal consistency (Cronbach’s alpha = 0.96 and 0.92) and test-retest reliability (ICC = 0.96 and 0.83). The convergent construct validity of Short-FES-I has been confirmed for previous falls, symptoms of depression, general disability, low quality of life; and physiocaloric impairment [29]. A score ≥10 on the Short-FES-I scale has been determined to indicate a fear of falling [29].

### Fall occurrence

The number of falls during the hospital stay, the time of each fall and any consequences will be recorded. Falls (and their dates) within the 6 months prior to hospitalization will also be collected.

### Length of stay

The length of stay in days and hours on the acute geriatric or post-emergency medicine ward will be calculated.

In view of the fact all patients will be evaluated during their hospital stay, loss of follow up should be minimal.

### Intervention

After the evaluation on day 1, patients in the experimental arm will wear the non-slip socks and patients in the control arm will remain barefoot for their entire hospital stay, even if a relative brings them a pair of suitable shoes. A sign will be posted in the patients’ rooms to ensure that this is respected. All patients will undergo rehabilitation as part of usual care. After the evaluation on D8, they will continue to receive usual care according to their pathology and needs, however they will remain either barefoot or with non-slip socks according to their group allocation.

### Management

An electronic Case Report Form (eCRF) will be created for each patient. All information required by the protocol will be provided in the eCRF. The eCRF will include the data needed to confirm compliance with the protocol and all data needed for statistical analyses; it will allow identification of any major deviations from the protocol. The person(s) responsible for completing the eCRF (investigator, clinical research assistant etc.) will be defined and identified in the task delegation form (kept in the investigator workbook). At the end of the study, the investigator will sign the eCRFs to certify the conformity of the data collected.

Data monitoring will be carried out by an employee of the department of the clinical research unit of Vendee Hospital to ensure that the electronic Case Report Forms (eCRF) are completed accurately. If an inspection or audit is conducted, the sponsor and/or the participating center will provide access to the data to the inspectors/auditors. A Clinical Research Associate (CRA) will regularly visit the site to carry out quality control of the data reported in the eCRF and to verify that the research is conducted according to the protocol. The monitoring plan has been defined and adapted to the estimated level of risk for patients who are suitable for research.

The protocol will be monitored to ensure that:

- informed consent has been received from each participant
- the clinical center has complied with the requirements of the protocol
- the data are complete and accurate
- all original patient information and documentation are present and complete.

The protocol is considered to have a Risk Level A (‘low or negligible risk’): no serious adverse events are expected. In accordance with article L1123-10 of the French Public Health Code, the applicable vigilance measures are those put in place for the practice of care.

Any substantial amendments will be sent by the sponsor to the ethics committee in accordance with French law. After authorization from the ethics committee, the sponsor will communicate the amended protocol to the investigators. Substantial changes will be added on ClinicalTrials.gov

A person’s participation may be terminated prematurely for the following reasons:

- Withdrawal of consent by the patient: patients will be able to withdraw their consent and request to be removed from the study at any time for any reason.
- Death.

Confidentiality of the identities of the patients who participate in the study will be guaranteed using a coding system. The presentation of the results of the research will exclude any direct or indirect identification.

The study has been registered on the open-access Clinical trials website. The results will be published in a journal and presented at a congress.

## Statistical methods

All variables will be described overall and by group. Qualitative variables will be described using numbers and percentages, and quantitative variables using minima, maxima, means, standard deviations and medians and interquartile ranges. We will collect data on the EnnovClinical eCRF. R version 4.2.0 or later will be used to perform all data analyses.

Gait speed during the 10m timed walk at D1’will be compared with speed during the dual task walk, as well as with speed during the imagined walk using a multiple linear regression taking into account the baseline value at D1. The same type of model will be used for comparing D8 evaluations.

The proportions of patients with a score ≥10 on the FESI (indicating a fear of falls) on D1 and D8 will be compared using the Chi-square test.

The length of stay will be compared between groups using the Student’s t-test.

The fall rate during the hospital stay will be estimated. Any consequences will be described.

All missing data and the reason for the missing data will be described in each group. For the primary outcome, if missing data are present at D8, they will be imputed by the patient’s baseline value (D1).

If more than 10% of missing data are observed for this outcome, a sensitivity analysis on the imputation method will be performed: a multiple imputation method will be applied.

### Study timeline

Recruitment began on 1 March 2022 and will continue until 1 March 2023. Data analysis will be carried out in September 2023 and the manuscript will be completed in December 2023.

### Ethics

Authorisation for the study was given on 3 December 2021 Comité de Protection des Personnes de Tours—Région Centre—Ouest 1. Hôpital Bretonneau–Centre Hospitalier Régional Universitaire de Tours 2, bd. Tonnellé - 37044 TOURS Cedex 9.

The approval number for a study involving the human being is 21.02015.202105-MS01.

The consent of the patients is verbally consent.

## Discussion

The aim of this study is to evaluate the benefit of non-slip socks on the maintenance and/or recovery of gait capacities in a geriatric hospitalized population. This study is the first RCT to specifically evaluate the impact of non-slip socks on several aspects of gait and falls [8]. The results should provide insight into whether non-slip socks are safe and effective for patients who arrive at the hospital with no footwear or with inadequate footwear.

The main limitation of this study is that it cannot be double blind, this is a classic limitation in rehabilitation studies. The main limitation of this study is that it cannot be double blind, this is a classic limitation in rehabilitation studies. Other limitations are identified and could be clarified in a future study based on the results of this study, such as the impact of medications, the age of the patients, or the medium- and long-term benefit of this intervention.

## Conclusion

This randomized controlled trial should provide clinicians with a scientific rational for the recommendation, or not, of the use of non-slip socks for older patients in acute care hospitals.

## Supporting information

S1 File (DOCX)

S2 File (DOCX)

S1 Checklist (DOCX)
