# Supplementary material for: Evaluation of the impact of non-slip socks on the motor recovery of elderly people in acute care hospitals: Protocol for a randomized, controlled trial study
Source: PLoS One. 2023 May 1;18(5):e0283226. doi: 10.1371/journal.pone.0283226 (PMC10150981; doi:10.1371/journal.pone.0283226)
Supplement: S1 File — (DOCX) [file pone.0283226.s001.docx]

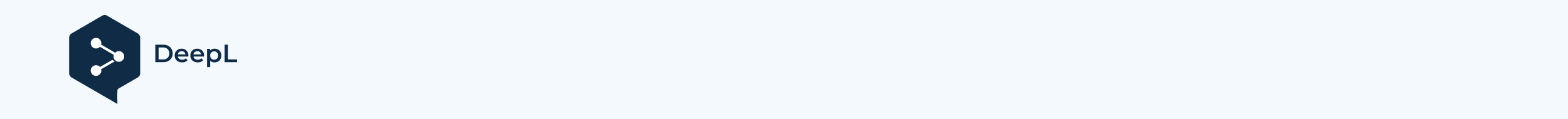


Subscribe to DeepL Pro to edit this document.
Visit [www.DeepL.com/pro](https://www.deepl.com/pro?cta=edit-document) for more information.

**ChARM Protocol**

**Registration No**. 2021-A00349-32

**Ref :** CHD 20-0059

**"Evaluation of the impact of non-slip socks on motor recovery in the elderly**

**An open, single-center, randomized, controlled pilot study**

**Coordinating Investigator:**

Thomas Rulleau,

Research engineer - physiotherapist

Vendée Departmental Hospital Centre

Geriatric Short Stay Service and Clinical Research Unit

[thomas.rulleau@chd-vendee.fr](mailto:thomas.rulleau@chd-vendee.fr)

**Methodologist***:*

**Lucie PLANCHE**

Vendée Departmental Hospital Centre

Clinical Research Unit

[lucie.planche@chd-vendee.fr](mailto:lucie.planche@chd-vendee.fr)

**Sponsor:**

**Vendée Departmental Hospital Center**Clinical Research Unit
Boulevard Stéphane MOREAU
85 925 LA ROCHE SUR YON Cedex 09

Tel : 02 51 44 65 72
Fax : 02 51 44 65 85

Signature page

**SIGNATURE OF THE PROMOTER**

| The sponsor agrees to conduct this study in accordance with all applicable laws and regulations that may apply to the research and in accordance with the protocol. | | |
| --- | --- | --- |
| **Name and position of signing official :**  **For the promoter and by delegation of the Director General, the Director of Medical Affairs and Research** | **Date:** | **Signature:** |

**SIGNATURE OF INVESTIGATOR S/ Qualified Person**

| I have read all the pages of the protocol of the clinical study for which the CHD Vendée is the sponsor. I confirm that it contains all the information necessary for the conduct of the trial. I undertake to carry out the study in accordance with the protocol and the terms and conditions defined therein. I agree to conduct the study in compliance with :   - the principles of the "Declaration of Helsinki", - the legislative and regulatory provisions of the Public Health Code applicable to category 2 RIPHs, as well as the associated application texts   I also agree that the investigators and other qualified members of my team will have access to this protocol and to the documents related to the conduct of the study to enable them to work in accordance with the provisions of these documents. | | | |
| --- | --- | --- | --- |
| **Coordinating Investigator** | **Name:**  Thomas RULLEAU | **Date:** | **Signature:** |
| **Principal Investigator/Qualified Person** | **Name and Institution:**  Thomas RULLEAU | **Date:** | **Signature:** |

LIST OF ABBREVIATIONS

| ANSM | National Agency for the Safety of Medicines and Health Products |
| --- | --- |
| ARC | Clinical Research Associate (monitor) |
| BPC | Good Clinical Practice |
| PPC | Committee for the Protection of Persons |
| CNIL | National Commission for Information Technology and Civil Liberties |
| CRF | Case Report Form (observation booklet) |
| eCRF | Electronic Case Report Form |
| MR | CNIL Reference Methodology |
| TEC | Clinical Study Technician |

Table of Contents

[Signature page 2](#_Toc68769248)

[LIST OF ABBREVIATIONS 3](#_Toc68769249)

[Table of Contents 4](#_Toc68769250)

[INTRODUCTION 6](#_Toc68769251)

[1. Rationale for the study 7](#_Toc68769252)

[1.1. Positioning of the research 7](#_Toc68769253)

[1.2. Benefits and risks for research participants 9](#_Toc68769254)

[2. Objectives and judging criteria 11](#_Toc68769255)

[2.1. Objective and primary endpoint 11](#_Toc68769256)

[2.2. Objectives and secondary endpoints 11](#_Toc68769257)

[3. Study population 12](#_Toc68769258)

[3.1. Description of the population 12](#_Toc68769259)

[3.2. Inclusion criteria 12](#_Toc68769260)

[3.3. Criteria for non-inclusion 12](#_Toc68769261)

[4. DESIGN AND STUDY PROCESS 13](#_Toc68769262)

[4.1. Study schedule 13](#_Toc68769263)

[4.2. General research methodology 16](#_Toc68769264)

[4.3. Scheme of the study 17](#_Toc68769265)

[4.4. Description and justification of the treatment plan/method studied 18](#_Toc68769266)

[4.5. Description of the evaluation and data collected 18](#_Toc68769267)

[4.6. Identification of all source data not in the medical record 20](#_Toc68769268)

[4.7. Rules for terminating a person's participation 20](#_Toc68769269)

[4.8. INDEMNIFICATION 21](#_Toc68769270)

[5. SAFETY ASSESSMENT 22](#_Toc68769271)

[6. Data Management and Statistics 23](#_Toc68769272)

[6.1. Collection and processing of study data 23](#_Toc68769273)

[6.2. Statistics 24](#_Toc68769274)

[7. Administrative and regulatory aspects 27](#_Toc68769275)

[7.1. Right of access to source data and documents 27](#_Toc68769276)

[7.2. Data confidentiality 27](#_Toc68769277)

[7.3. Monitoring of the study 27](#_Toc68769278)

[7.4. Inspection / Audit 28](#_Toc68769279)

[7.5. delegation of tasks 28](#_Toc68769280)

[7.6. declaration to the competent authorities 28](#_Toc68769281)

[7.7. Amendments to the protocol 28](#_Toc68769282)

[7.8. Computerized data and submission to the CNIL 29](#_Toc68769283)

[7.9. Patient information 29](#_Toc68769284)

[7.10. Financing and insurance 29](#_Toc68769285)

[7.11. Rules for publication 29](#_Toc68769286)

[7.12. Archiving of source data 30](#_Toc68769287)

[8. Bibliographic references 31](#_Toc68769288)

[List of appendices 35](#_Toc68769289)

[8.1. Appendix 1: Short-Falls Efficacy Scale International 36](#_Toc68769290)

[8.2. Appendix 2: Confusion Assessment 37](#_Toc68769291)

[8.3. Appendix 3: Medline Anti-Slip Socks Instructions 38](#_Toc68769292)

INTRODUCTION

With advancing age, many patients lose their autonomy. This loss of autonomy can be increased during hospitalization, by the effect of the pathology itself, or by the effect of hospitalization. The objective of the rehabilitators is to set up tools to preserve the motor capacities of the patients, through rehabilitation sessions and/or with adequate equipment. In particular, they make sure that walking aids and footwear are adapted to the patient's clinical condition.

However, senior patients arriving in the medical department do not always have adequate footwear. The teams taking care of them, including the physiotherapists, are confronted with the choice of keeping the entrance shoe or proposing to the patients to walk barefoot. The choice to keep the usual footwear is the most frequent.

For some time now, it has been possible to offer non-slip socks for walking around their wards. However, there is a controversy in the scientific literature about the usefulness of these devices. In particular, it is impossible to transpose the results of studies to judge the interest of these socks in the senior subject.

The objective of this study is to evaluate the interest of anti-slip socks on motor recovery.

# Justification of the study

## Positioning of the research

### Rational

In 2012, people aged 65 or older represented 17.1% of the population (16% in 2002), half of which is represented by those aged 75 or older. There has been an increase of 45% in 20 years (INSEE, 2012). In 2015, in France, 2.5 million senior citizens are in loss of autonomy (INSEE, 2017). Among seniors aged 75 and over, 8.8% live in institutions (INSEE, 2017). The projection for 2050 would be 4 million seniors with a loss of autonomy, or 16.4% of seniors (INSEE, 2017) ..

Frequently, during a pathology leading to hospitalization, the aging of sick or frail people undergo a physical deconditioning. This deconditioning is a psychophysiological process leading to physical inactivity. It is defined "as an amplifier of vulnerability causing situations of dependence and an altered [quality of life](http://blogensante.fr/2013/09/02/definir-la-notion-de-qualite-de-vie/)**" (Préfaut & Ninot, 2003, p. 3).** (Préfaut & Ninot, 2009) **.** Muscle mass, strength and power decrease with age, and by the age of 80, older people have lost half their initial muscle mass (Professional Associations for Physical Activity, Sweden, 2010)

Figure 1: The vicious cycle of deconditioning according to Hadjistavropoulos et al (2011)

According to Hadjistavropoulos et al.2011)a vicious circle of deconditioning exists in the senior subject. The fall leads to a fear of falling, which leads to a restriction of activity, which leads to a functional decline, which leads to a decrease in stability, which leads to an increase in the risk of falling (**Error! Source of the reference not found.**).

When they arrive in short-stay wards, patients may be tired and/or confused, depending on the reason for their hospitalization. To this physical deconditioning can be added a deficit of stimulation which will be an aggravating factor, in particular in the first days of hospitalization. Physiotherapists will therefore be called upon.

Physiotherapists will ensure that seniors have good motor skills. Walking, transferring, moving around in their living space or carrying out activities of daily living is essential to maintain a quality of life in the living space of their choice. (The evaluation of motor skills will be developed later). An important skill for older people, in addition to the intention to move, is motor planning. Motor planning is the necessary step to build the basis of the motor program. Among many other possible movements due to the redundancy of the musculoskeletal system, the subject chooses a "typical movement". This motor planning seems to be impaired in some frail elderly patients (Kubicki & Mourey, 2015). In this mission of recovery of motor capacities, the physical therapist must restore as soon as possible to the patients a safe (self-evaluation) and secure (physical therapist evaluation) walking. For this, and according to the assessment of the physiotherapist, different technical aids exist (Temfemo & Ahmaidi, 2018). These aids include the simple cane, the tripod cane, English canes, walkers with or without wheels etc. The physical therapist will also need to pay attention to footwear.

Indeed, ill-fitting footwear may be experienced as unsafe and/or unsupportive by the team or the patient. Luk et al. (2015) offered a narrative review on fall prevention in older adults. They advocate fall prevention interventions such as exercise, environmental modifications, or medication review. These authors also propose to focus on the footwear of patients. They report proposals to avoid high heels and to use non-slip shoes based on other work (McKiernan, 2005; Menant et al., 2008).

Footwear worn by elderly subjects on geriatric wards has also been studied by several teams. Vass et al. (2015) examined and described the type of footwear worn by elderly patients in the hospital. They show that many patients wear footwear with insufficient structure to promote optimal stability or gait. Among the different types of footwear, they find non-slip socks for subjects at home, in institutions, or in hospitals (Menant et al., 2008; Vass et al., 2015). A recent narrative review looked at the use of non-slip socks to prevent falls in elderly patients (Hartung & Lalonde, 2017). In their conclusion, the authors do not recommend the use of socks for patients coming from home with limitations on the selected studies. An important limitation is the external validity of the studies, stemming from basic research for a part. The other part, from clinical research, does not specifically concern our reference population. As a result, it is not possible for us to recommend or not to recommend the wearing of non-slip socks in senior hospitalized patients. However, the question arises under specific conditions that we will detail.

In clinical practice, in the short-stay departments of many hospitals, including the CHD-Vendée, patients arrive from the emergency room without adapted footwear or without footwear at all. The emergency situation has led them to the hospital without giving them time to take their belongings. In direct hospitalizations, a re-evaluation of footwear is also often necessary to adapt the footwear to the patient's situation. Other patients, especially those with major cognitive disorders who seem to be most at risk, regularly forget their shoes during the day and at night. In the end, autonomy seems to be impaired by the lack of adapted shoes. During the care process (washing, dressing, etc.), the caregivers will move the patient to the chair rather than having him/her walk to the bathroom. In rehabilitation, the practitioner may choose not to take charge of a patient, or to propose a partial takeover to avoid the risk of inadequate footwear. The patient may be offered to walk alone outside of rehabilitation.

### The objective of the study

In the end, we see a possible clinical interest of anti-slip socks. The scientific literature presents studies with methodological limitations, but above all a different population than the one we are interested in. It is therefore not currently possible to judge the specific interest of anti-slip socks in an elderly hospitalized population.

Evaluating the interest of these socks could allow them to be recommended with solid data. Preventing this iatrogenic loss of autonomy would have a positive impact on the motor skills of patients, a quicker and easier return home, and a reduction in health costs.

We would therefore like to know the interest of wearing anti-slip socks in the management of these patients, and more particularly on the main objective of rehabilitation in geriatric physiotherapy: motor function.

We believe that wearing non-slip socks improves motor recovery in patients.

## Benefits and risks for individuals involved in research

### Benefits

#### Individual benefit

Improved motor support

Improved practices with the use of non-slip socks

#### Collective benefit

Improvement of the motor care of the senior subject allowing a lower cost for the society thanks to a shorter duration of stay,

Improved motor management for faster discharge

### Foreseeable constraints and risks

Constraints:

For both groups, the only constraint added by the research is the taking of tests and the filling out of the questionnaire specific to the research.

For the experimental group, the additional constraint is the wearing of non-slip socks.

Foreseeable risks:

There is no increased risk by wearing non-slip socks in senior patients.

### Benefit/risk balance

This study is qualified as interventional research with minimal risks and constraints as defined in article L1121-1 of the Public Health Code. On the basis of all the information mentioned above, the benefit-risk balance of this study is evaluated favorably.

# Objectives and judging criteria

## Objective and primary endpoint

### Main objective

Evaluating **the impact of anti-slip socks on motor recovery** in the elderly during hospitalization

### Primary endpoint

Evolution of **walking speed at the patient's preferred speed over 10m** between D1 (start of physiotherapy treatment) and D8. Two tests will be performed at each evaluation, the average of the two tests will be used.

## Objectives and secondary endpoints

### Secondary Objective(s)

1. Evaluate the impact of anti-slip socks on short-term motor skills
2. Evaluate the impact of non-slip socks on motor planning in the short term and during hospitalization
3. To assess the impact of wearing socks on the ability to manage the dual task in the short term and during hospitalization
4. To evaluate the evolution of fear of falling with the use of socks in the short term and during hospitalization
5. Evaluate the impact of socks on the presence of falls
6. Length of stay in a geriatric or post-emergency medical facility

### Secondary endpoint(s)

1. Evolution of walking speed over 10m between Day 1 (beginning of physiotherapy treatment) and Day 1' ("after a 3-minute walk with the shoe assigned following randomization"),
2. Evolution of the isochrony between the speed of the walk actually performed and the speed of the imagined walk over 10m at D1, D1' and D8. The average of two tests of each modality will be used.
3. Evolution of isochrony between walking speed and double-task walking speed over 10m at D1,D1' and D8 (Beauchet et al., 2010; Menant et al., 2014). The average of two tests of each modality will be used.
4. Total score > 10 on the Short-Falls Efficacy Scale International at D1, D1' and D8
5. Presence of fall(s) and its consequences during hospitalization
6. Length of stay calculated from the beginning of the hospitalization to the end of the hospitalization

# Study population

## Description of the population

This study is aimed at patients admitted to the CHD Vendée in Post-Emergency Medicine and Geriatric Short Stay who require physiotherapy.

## Inclusion criteria

- Patient in Geriatric Short Stay or Post-Emergency Medicine,
- Patient 75 years and older,
- Patient requiring physical therapy,
- Patient with a minimum of 7 days of physical therapy
- Patient arrived with unsuitable footwear (assessment at clinician's discretion: no back brace, unsuitable size etc.) or without footwear,
- Patient able to walk at least 10 m with or without technical assistance,
- Patient with oral consent
- Patient with social security coverage.

## Criteria for non-inclusion

- Inability to understand or perform study-specific clinical tests
- Patients with a history of dementia assessed prior to their hospitalization, with an MMSE score ≤ 22/30
- Blind patient
- Patient under guardianship or curatorship
- Patient participating in an interventional clinical research protocol that may alter the assessments of this protocol.
- Patient unable to wear socks or to walk barefoot (need for compression socks or stockings, wound, too much edema, other)

The investigator/qualified person, or a person designated by the investigator/qualified person, should maintain a list of patients not included throughout the study. This list should include all patients from the population who do not meet the eligibility criteria. The list should also indicate, for each patient, the reason for non-inclusion in the study.

# DESIGN AND STUDY PROCESS

## Study schedule

### Inclusion and Randomization

Patients arrive in the department and are assessed by the doctor who prescribes physiotherapy if necessary.

The physiotherapist takes note of the prescription on the next working day. The physiotherapist performs a routine analysis of the patient's footwear. If the footwear is not suitable or if the patient is barefoot, the physiotherapist checks the patient's eligibility criteria. If the patient meets these criteria, he/she is informed of the study. If the patient gives oral consent, the physiotherapist will perform the **randomization**.

**Randomization arm:**

- **Control arm: usual care with bare feet.**
- **Experimental arm: specific management with anti-slip socks.**

### Baseline: J1 Tests

The physiotherapist assesses the patient during the first session's diagnostic assessment and performs the *Confusion Assesment Method* if it has not been done in routine practice.

A Visual Analog Fatigue Assessment will be performed prior to clinical testing.

In the framework of the research, the **J1** tests will be performed barefoot:

1. Patient's walking speed over 10 m (2 times)
2. Walking speed in a dual-task situation over 10 m (2 times)

Two other less common but validated assessments in this population will be performed.

1. Imagined walking speed over 10 m (2 times)

The average of each of the two tests will be used for items 1 to 4.

All these tests represent a duration of about 10 minutes for the patient.

1. Short-Falls Efficacy Scale International Questionnaire

Depending on the randomization arm, the physical therapist will put non-slip socks on the patients in the experimental arm and leave the patients in the control arm barefoot. He will then encourage the patient to walk for 3 minutes.

### Follow-up J1'.

After these 3 minutes of walking with or without the socks, depending on the randomization arm, and in order to evaluate the immediate effect of wearing anti-slip socks on motor skills, the patient will perform the same tests as at D1 in the same order.

A Visual Analog Fatigue Assessment will be performed before the following clinical tests:

1. Patient's walking speed over 10 m (2 times)
2. Walking speed in a dual-task situation over 10 m (2 times)
3. Imagined walking speed over 10 m (2 times)

The tests will be performed by the patient with bare feet (control arm) or with the anti-slip socks (experimental arm).

### Procedure for the care of the patient during the stay

Following this second evaluation and during the entire duration of hospitalization, socks will be left on the patients in the experimental arm, and the patients in the control arm will remain barefoot. A poster will be posted in the patients' rooms to inform the care teams so that the randomization arm is respected.

Between D1' and D8', the patient will be followed in rehabilitation according to the pre-established plan based on the conclusions of the physiotherapy diagnostic assessment (BDMK). The patient will have 3 sessions, the first one including the assessment at D1.

### Follow-up J8

At D8, in order to evaluate the effect of the socks on motor preservation/recovery, the patient will be re-evaluated under the same conditions as at D1, i.e., barefoot, by the same tests as at D1 and in the same order.

A Visual Analog Fatigue Assessment will be performed before the following clinical tests:

1. Patient's walking speed over 10 m (2 times)
2. Walking speed in a dual-task situation over 10 m (2 times)
3. Imagined walking speed over 10 m (2 times)
4. Short-Falls Efficacy Scale International Questionnaire

**Study schedule**

| **Actions** | J1 | D1' (after 3 minutes of free walking) | J8 | Discharge from hospital |
| --- | --- | --- | --- | --- |
| Patient information | X |  |  |  |
| Collection of Informed Consent | X |  |  |  |
| Verification of inclusion and non-inclusion criteria | X |  |  |  |
| Randomization | X |  |  |  |
| *Confusion Assessment Method* | X |  |  |  |
| EVA fatigue | X | X | X |  |
| History (fall, MMSE etc.) | X |  |  |  |
| Type of technical aid | X | X | X |  |
| Clinical examination | X |  | X |  |
| Tests:   - Walking speed at the patient's preferred speed over 10m (x2), - Walking speed in double task over 10m (x2), - Imagined walking speed over 10m (x2), | X | X | X |  |
| Short-FESI Questionnaire | X |  | X |  |
| Length of stay in a geriatric or post-emergency medical facility |  |  |  | X |

.

## General research methodology

The research has the following characteristics:

- Open study,

- Monocentric (CHD Vendée)

- Controlled,

- Of superiority,

- Randomized

Duration of inclusion: 18 months

Duration of participation: 7 days

Duration of the research: 19 months maximum

Number of patients to be randomized: 50

## Scheme of the study

Indication and prescription

Eligibility criteria met

Consent and inclusion

Randomization

Assessment D1:

Barefoot evaluation

1st arm:

Non-slip socks

2nd arm:

Barefoot

Evaluation D1' at 3 minutes

Evaluation with socks

anti-skid

Evaluation D1' at 3 minutes

Barefoot evaluation

Rehabilitation according to the diagnostic assessment

masso-kinésithérapique

Rehabilitation according to the diagnostic assessment

masso-kinésithérapique

Evaluation J8

Barefoot

## Description and justification of the treatment plan/method studied

The objective of the study is to evaluate the effect of wearing non-slip socks on motor recovery. We have therefore chosen a design where we will be able to evaluate the evolution of motor skills between a control group and the experimental group wearing socks.

The non-slip socks used in this study (Appendix 3) will be those used in our usual practice.

These socks from Medline have pimpled strips on both outer sides for continuous grip, even when the sock is turned. They have an absorbent terry cloth interior to keep feet dry. They are latex-free to avoid allergies.

Socks will be changed daily to maintain good foot hygiene. The change will be done by the medical staff at the patient's bed.

## Description of the evaluation and the data collected

### Walking speed

Gait speed is a common physical therapy test used to assess motor function. It is simple to use even for patients with cognitive and confusional disorders. The gait speed of patients over 80 years of age is 0.943m/seconds ± 0.091 (Bohannon & Williams Andrews, 2011; Menant et al., 2014)

The best initial estimates of small significant changes are near 0.05 m/s for walking speed. (Perera et al., 2006)

Substantial changes are on the order of 0.10 m/s (Perera et al., 2006).

There are several modalities for performing the test, at usual walking speed or at fast walking speed. For the purpose of this protocol, we chose the patient's preferred speed walking modality (Bohannon & Williams Andrews, 2011).

Ground markings or studs will give the patient a visual cue. The patient stands in front of the 10m course, with visual cues to remind him/her.

The walking speed test will be performed twice. The instructions are:

"At my starting point, you will have to walk at your usual speed to pass the marker."

"Are you ready?"

### Walking speed in double task

The speed of walking in a dual task gives an indication of the cognitive reserve available (Lundin-Olsson et al., 1997). A cessation of walking or a decrease in walking speed of more than 30% is a predictive factor for falls (Beauchet & Berrut, 2006).

Markings on the ground or studs will provide a visual cue. The patient stands in front of the 10m course, with visual cues to remind him/her.

The walking speed test will be performed twice. The instructions are:

"At my starting signal, you will walk at your usual speed past the marker/plot on the ground. During this walk, you will have to name as many animals as possible.

"Are you ready?"

### The imagined walking speed

Human movement can be separated into 2 phases, a programming phase (anticipation) and an execution phase (Jeannerod, 2001). The concordance between planning and execution of movement can be reliably evaluated thanks to a simple test evaluating the temporal concordance between an imagined practice and a physical practice (Guillot et al., 2012). This concordance is translated into an Isochrony Index. Adapted to the imagined walking speed, it is a simple test aiming to evaluate the motor prediction capacities (Rulleau et al., 2015). Interestingly, the decrease in isochrony correlates with the decrease in gait speed during the dual task (Bridenbaugh et al., 2013)). Thus, it could mark a risk of falling due to poor anticipation of actual motor abilities.

Markings on the ground or studs will provide a visual cue. The patient stands in front of the 10m course, with visual cues to remind him/her.

The imagined walking speed test will be performed twice. The instructions are

"At my starting signal, you will have to imagine yourself walking at your usual speed to pass the marker/plot on the ground while remaining in your seat. When you imagine passing it, you will say "stop".

"Are you ready?"

### Fear of falling (Appendix 2)

The Short-Falls Efficacy Scale International (Short-FES-I) is a self-administered questionnaire completed by patients and composed of 7 items with 4 possible answers. It has excellent internal consistency (Cronbach's alpha = 0.96 and 0.92) and test-retest reliability (ICC = 0.96 and 0.83). The convergent construct validity of the Short-FES-I was confirmed for: prior falls; depressive symptoms; general disability; low quality of life; and physi-caloric impairment (Dewan & MacDermid, 2014).

A threshold for a fear of falling has been defined as >10 on the Short-FES-I scale (Dewan & MacDermid, 2014).

### The presence of a fall

The occurrence of a fall during the hospitalization, the time of the fall and its consequences, the total number of falls during the hospitalization, will be recorded.

Falls and dates of falls prior to hospitalization of less than 6 months will also be collected.

### Confusion Assesment Method (Appendix 3)

This tool is intended to be used by a caregiver, who looks for 4 groups of clinical signs (Haute Autorité de Santé, 2009; Kharat & Simonet, 2013) :

1. Sudden onset and fluctuating symptoms;
2. Inattention;
3. Disorganized thinking;
4. Disturbances of vigilance.

Diagnosis requires the presence of 3 of the 4 criteria. Criteria 1 and 2 are always required, accompanied by 3 or 4 (Kharat & Simonet, 2013; Appendix 3).

### Visual Analog Scale of Fatigue

The visual analog scale is commonly used in the assessment of pain (Hawker et al., 2011). In addition, other authors propose its use in the assessment of fatigue under the same modality (Hewlett et al., 2011).

The ruler is presented to the patient in these terms:

"This is a slider representing your fatigue, on the left no fatigue at all, on the right the maximum fatigue you can imagine. I'm going to ask you to move the slider to your current fatigue level.

The operator rates the fatigue from 0 to 100.

### Length of stay

The length of stay in days and hours in geriatric short stay or post-emergency medicine calculated between the beginning of the hospitalization and the end of the hospitalization will be listed.

## Identification of all source data not in the medical record

1. Confusion Assesment Method Scale at D1
2. Visual Analog Scale of Fatigue at D1, D1' and D8
3. Type of technical assistance at D1, D1', D8
4. Duration of the 10 m walk (2 times) performed at D1, D1' and D8
5. Duration of the double-task walk over 10 m (2 times) performed at D1, D1' and D8
6. Duration of the imagined 10 m walk (2 times) performed at D1, D1' and D8
7. Short-Falls Efficacy Scale International Questionnaire at D1, D1' and D8

## Rules for stopping a person's participation

### Criteria for premature termination of an individual's participation in research

An individual's participation may be terminated prematurely for the following reasons:

- Withdrawal of consent by the patient

Patients will be able to withdraw their consent and ask to leave the study at any time for any reason.

- Death.

### Monitoring and data collection schedule

Patients who will be exited from the study, will not continue the study schedule.

In case of withdrawal of consent by the patient, and without prejudice to the patient's right; the data already collected will be analyzed in accordance with the regulations.

However, no other examinations specifically foreseen by the protocol will be performed and no data will be collected.

The discharge of a patient from the study will not change the patient's usual management of his or her pathology.

### Criteria for stopping part or all of the research (excluding biostatistical considerations)

Part or all of the study may be stopped permanently or temporarily by decision of the ANSM, the CPP and/or the Study Sponsor.

In all cases:

- A written confirmation will be sent to the coordinating investigator of the study (specifying the reasons for premature termination*),*

-All patients in the study will be informed.

### Management of patients at the end of the research

Patients will be managed according to the usual practices of the department.

## INDEMNIFICATION

There is no compensation for participation in this study.

# SAFETY ASSESSMENT

As the study corresponds to a category 2 RIPH (Research Involving Human Subjects), the provisions applicable in terms of vigilance are those put in place in the practice of care and the use of products associated with the study in accordance with article L1123-10 of the Public Health Code. The investigator will therefore be responsible for reporting the occurrence of any adverse event according to the procedures applicable to the CHD Vendée.

# Data Management and Statistics

## Collection and processing of study data

### Data collection

One observation book (eCRF) will be created per patient. All information required by the protocol must be provided in the eCRF. It should include the data needed to confirm compliance with the protocol and all data needed for statistical analyses; it should identify major deviations from the protocol.

The person(s) responsible for filling in the eCRFs (investigator, CRA, etc.) must be defined and is/are identified in the task delegation form (kept in the investigator's folder).

At the end of the study, the investigator will sign the eCRFs to certify the compliance of the data collected.

### Data coding

By signing this protocol, the principal investigator/qualified person and all members of his or her team agree to keep confidential the identities of the patients who participated in the study.

The transmission of a person's data for research purposes will therefore only be possible if a coding system is applied; the presentation of the research results will exclude any direct or indirect identification.

The identification of patients will be done according to the order of inclusion of patients by a number automatically assigned by the Ennov Clinical software (eCRF) then completed by the initials of the patients (1st letter of the first name + 1st letter of the last name).

This code will be the only information that will appear on the eCRF and will allow the eCRF to be linked to the patient after the fact.

The investigator/qualified person is also required to code patient data on any documents he/she may have in his/her possession that are attached to the eCRF.

A correspondence table will be set up at the participating center. This table will be kept in a secure place by the principal investigator/qualified person of the center and will contain the patient code and his nominative data in order to be able to trace back to the patient file in case of missing or erroneous data. No clinical data will be collected in these correspondence tables.

### Data processing

The collection of clinical data will be based on the implementation of a database and the creation of data entry masks similar to the observation book in accordance with the protocol and regulations currently in force.

## Statistics

Software

The analyses will be carried out under the software R version 3.5.1

### Description of planned statistical methods, including schedule of planned interim analyses

All variables will be described globally and by group. The description will include the numbers and percentages of the modalities for the qualitative variables and the minimum, maximum, average, standard deviation and median for the quantitative variables.

The set of criteria gait speed, isochrony between imagined and executed gait speed, isochrony between normal gait speed and dual-task gait will be compared using linear models taking into account the baseline value J1.

As the evaluation conditions between D1' and D8 are different, the models evaluating the evolution at D1' and at D8 will be independent.

Fear of falling will be assessed using the Falls Efficacy Scale International questionnaire. Fear of falling is defined with a cut-off score of 10 on this questionnaire.

The number of patients with its percentage having fear of falling will be presented and compared at D1' and D8 using a Chi-square test.

Length of stay will be compared using a Student's t test.

The presence of falls and their consequences will be described during the hospital stay.

### Statistical justification of the number of inclusions

Gait speed is a common physical therapy test used to assess motor function. It is simple to use even for patients with cognitive and confusional disorders. The gait speed of patients over 80 years of age is 0.943m/seconds ± 0.091 (Bohannon & Williams Andrews, 2011; Menant et al., 2014)

According to Perera et al. an improvement in walking speed of 0.10 m/s is considered clinically relevant (Perera et al., 2006).

In the context of this study, we therefore retain a difference of 0.10m/s to be highlighted between the 2 groups and a standard deviation of the difference fixed at 0.10.

Based on these assumptions and for an alpha risk of 5% and a power of 90%, a total of 46 patients are required. In order to guarantee the power of the study, 50 patients will be randomized.

### Expected level of statistical significance

The alpha risk is set at 5%.

### Statistical criteria for stopping the research

NA

### Method of accounting for missing, unused or invalid data

All missing data and their reasons will be described in each group.

For the primary endpoint, if missing data are present at D8 they will be imputed by the patient's baseline value (D1).

If for this criterion, more than 10% of missing data are observed, a sensitivity analysis on the imputation method will be performed: a multiple imputation method will be applied.

### Managing changes to the initial strategy analysis plan

NA

### Selection of individuals to be included in the analyses

The main analysis will be performed on the Intent-to-Treat (ITT) population, i.e. on all randomized patients.

A complementary analysis will be performed on the Per Protocol (PP) population including randomized patients for whom no major protocol deviations have been identified.

A data review meeting will be held to review and define the major criterion or not for each of the deviations.

### Randomization

Randomization will not be stratified

It will be carried out in a 1:1 ratio and will be done in blocks.

The randomization will be done in Ennov Clinical by connecting to the website: https://nantes-lrsy.hugo-online.fr/EnnovClinical/. The connection will be done thanks to a login, a password and a study number, delivered by the data-manager of the Research Unit of the CHD of La Roche sur Yon. The following information must be filled in:

-First initial of the name,

-First initial of the first name,

-Month and year of birth,

Compliance with inclusion and non-inclusion criteria (yes/no),

Randomization will be performed by the physiotherapist or other authorized person after confirmation of the patient's inclusion in the study and oral consent has been obtained. Randomization will be performed before the D1 assessments. The inclusion number will be assigned automatically during randomization. An email confirmation will be sent to the person who performed the randomization and to all persons involved.

The randomization list will be carried out by the biometrics team of the Research Unit of the CHD of La Roche sur Yon. An explanatory guide of the randomization will be available online under Ennov Clinical.

# Administrative and regulatory aspects

## Right of access to source data and documents

The investigators will make available to the persons in charge of monitoring, quality control or auditing the research, the documents and individual data strictly necessary for this control, in accordance with the legislative and regulatory provisions in force (articles L.1121-3 and R.5121-13 of the Public Health Code).

## Data privacy

Persons with direct access will take all necessary precautions to ensure the confidentiality of information relating to the persons who have access, particularly with regard to their identity and the results obtained.

These persons, as well as the investigators themselves, are subject to professional secrecy (according to the conditions defined by articles 226-13 and 226-14 of the penal code).

During or at the conclusion of the research, the data collected on the individuals involved in the research and transmitted by the stakeholders will be pseudonymized.

Under no circumstances should the names of the persons concerned or their addresses appear in clear text.

Only the first letter of the person's last name and the first letter of his or her first name and year of birth will be recorded, along with a coded number specific to the study indicating the order of inclusion of patients*.*

## Monitoring of the study

Monitoring will be carried out by the Promotion Department of the Research Directorate. A Clinical Research Associate (CRA) will regularly visit each site to check the quality of the data reported in the observation books.

The monitoring plan is defined and adapted to the level of risk estimated for the patient undergoing the research. It will be monitored in the following manner:

Risk A: low or negligible foreseeable risk

On-site monitoring visits will be arranged by appointment with the investigator/qualified person. CRAs will need to have access to:

- the data collection books of the included patients,

- patient medical and nursing records,

- the investigator binder.

## Inspection / Audit

As part of this study, an inspection or audit may take place. The sponsor and/or participating center must be able to provide access to the data to the inspectors or auditors.

## delegation of tasks

The principal investigator of the research site establishes and maintains a task delegation form that specifies the respective tasks that he or she delegates to members of his or her team in the study, according to their competence.

Each investigator's collaborator prepares a dated and signed curriculum vitae (CV) that is kept up to date.

The investigator ensures that the collaborators to whom he/she delegates tasks in the study have the appropriate competence for those tasks. The investigator remains responsible for the conduct of the research at the site.

## declaration to the competent authorities

The sponsor undertakes to submit the study project for prior authorization by a Comité de Protection des Personnes (CPP). The information communicated concerns, on the one hand, the modalities and nature of the research and, on the other hand, the guarantees planned for patients participating in this study. The sponsor submits the curriculum vitae of the principal investigator of the research site to the CPP for its opinion.

.

This protocol will also be reported to the ANSM.

## Amendments to the protocol

Requests for substantial modifications will be sent by the promoter to the CPP concerned for its opinion, in accordance with the law in force and its implementing decrees.

The amended protocol must be updated, dated and signed.

The information letters and the oral consent form will need to be modified as necessary.

## Computerized data and submission to the CNIL

The data collected in this study is for scientific research purposes, for the public good.

This study falls within the framework of the "Reference Methodology" MR-001 registered, for the CHD Vendée, under n°2060482 v 0 for the following reasons

- Collection of health data for research purposes

- Obtaining the opinion of a CPP to start the research

- Use of pseudonymized data

- Individual information of the persons concerned

- Access to data only by professionals (health care and sponsor) involved in the study.

The fact that this study falls within the scope of MR001 and the reasons for it will be notified in the sponsor's treatment registry.

## Patient information

### Informed consent oral

The investigator/qualified person undertakes to obtain the free, informed and express consent of the person, obtained orally, after having provided information on the protocol. The investigator/qualified person will give the person a copy of the information note. The person can only be included in the study after having read the information note and given oral consent after having had time to reflect, if necessary.

The patient's information and consent to participate in the research should be noted in the patient's medical record.

A record of the collection of the patient's express oral consent will be kept in the study documents.

## Financing and insurance

The promoter ensures the financing of the study and subscribes to an insurance policy guaranteeing the pecuniary consequences of its civil liability, in accordance with the regulations.

## Rules for publication

The study will be registered on the Clinical trial open access website before the inclusion of the 1^er^ patient in this study.

The study may not be the subject of any written or oral commentary without the agreement of the promoter; all information communicated or obtained during the course of the study belongs by right to the CHD Vendée, which may freely dispose of it.

All information resulting from this study is considered confidential, at least until appropriate analysis and monitoring by the sponsor, study coordinator and statistician is completed.

The scientific papers and reports corresponding to this study will be produced under the responsibility of the study coordinator.

The study coordinator will be the principal signatory of the paper and the editor of the documents, and will necessarily be listed as the first or last author. He/she may delegate this task to another person.

The coordinating investigator establishes the list of authors. The investigators will be cited in proportion to the number of patients recruited. The statistician of the study will also be cited.

Likewise, publications of ancillary results will include the name of the person who performed the ancillary work as well as the names of all other persons involved in the ancillary work.

All publications, abstracts or presentations including the results of the study must be submitted to the sponsor (CHD Vendée) for approval.

The rules of publication will follow international recommendations (N Engl J Med, 1997; 336:309-315). .

## Archiving of source data

The investigator/qualified person must retain all study information for at least 15 years after the study is completed.

At the end of the study, the investigator/qualified person will receive a copy of each patient's data from the sponsor.

No removal or destruction shall be made without the consent of the Developer. At the end of the 15 years, the Developer will be consulted for destruction. All data, documents and reports may be subject to audit or inspection.

# Bibliographic references

Beauchet, O., Annweiler, C., Assal, F., Bridenbaugh, S., Herrmann, F. R., Kressig, R. W., & Allali, G. (2010). Imagined Timed Up & Go test: A new tool to assess higher-level gait and balance disorders in older adults? *Journal of the Neurological Sciences*, *294*(1-2), 102-106. https://doi.org/10.1016/j.jns.2010.03.021

Beauchet, O., & Berrut, G. (2006). [Gait and dual-task: Definition, interest, and perspectives in the elderly]. *Psychology & neuropsychiatry of aging*, *4*(3), 215-225.

*Bilan social 2015 | ATIH Publication*. (2015). https://www.atih.sante.fr/bilan-social-2015

Bohannon, R. W., & Williams Andrews, A. (2011). Normal walking speed: A descriptive meta-analysis. *Physiotherapy*, *97*(3), 182-189. https://doi.org/10.1016/j.physio.2010.12.004

Bridenbaugh, Beauchet, Annweiler, Allali, Herrmann, F., & Kressig, R. W. (2013). Association between dual task-related decrease in walking speed and real versus imagined Timed Up and Go test performance. *Aging Clinical and Experimental Research*, *25*(3), 283-289. https://doi.org/10.1007/s40520-013-0046-5

Dewan, N., & MacDermid, J. C. (2014). Fall Efficacy Scale-International (FES-I). *Journal of Physiotherapy*, *60*(1), 60. https://doi.org/10.1016/j.jphys.2013.12.014

Guillot, Hoyek, Louis, & Collet (2012). *Understanding the timing of motor imagery: Recent findings and future directions*. *5*(1), 3-22.

Hadjistavropoulos, T., Delbaere, K., & Fitzgerald, T. D. (2011). Reconceptualizing the role of fear of falling and balance confidence in fall risk. *Journal of Aging and Health*, *23*(1), 3-23. https://doi.org/10.1177/0898264310378039

Hartung, B., & Lalonde, M. (2017). The use of non-slip socks to prevent falls among hospitalized older adults: A literature review. *Geriatric Nursing (New York, N.Y.)*, *38*(5), 412-416. https://doi.org/10.1016/j.gerinurse.2017.02.002

Haute Autorité de Santé. (2009). *Acute confusion in the elderly: Initial management of agitation*. Haute Autorité de Santé. https://www.has-sante.fr/jcms/c_819557/fr/confusion-aigue-chez-la-personne-agee-prise-en-charge-initiale-de-l-agitation

Hawker, G. A., Mian, S., Kendzerska, T., & French, M. (2011). Measures of adult pain: Visual Analog Scale for Pain (VAS Pain), Numeric Rating Scale for Pain (NRS Pain), McGill Pain Questionnaire (MPQ), Short-Form McGill Pain Questionnaire (SF-MPQ), Chronic Pain Grade Scale (CPGS), Short Form-36 Bodily Pain Scale (SF-36 BPS), and Measure of Intermittent and Constant Osteoarthritis Pain (ICOAP). *Arthritis Care & Research*, *63 Suppl 11*, S240-252. https://doi.org/10.1002/acr.20543

Hewlett, S., Dures, E., & Almeida, C. (2011). Measures of fatigue: Bristol Rheumatoid Arthritis Fatigue Multi-Dimensional Questionnaire (BRAF MDQ), Bristol Rheumatoid Arthritis Fatigue Numerical Rating Scales (BRAF NRS) for severity, effect, and coping, Chalder Fatigue Questionnaire (CFQ), Checklist Individual Strength (CIS20R and CIS8R), Fatigue Severity Scale (FSS), Functional Assessment Chronic Illness Therapy (Fatigue) (FACIT-F), Multi-Dimensional Assessment of Fatigue (MAF), Multi-Dimensional Fatigue Inventory (MFI), Pediatric Quality Of Life (PedsQL) Multi-Dimensional Fatigue Scale, Profile of Fatigue (ProF), Short Form 36 Vitality Subscale (SF-36 VT), and Visual Analog Scales (VAS). *Arthritis Care & Research*, *63 Suppl 11*, S263-286. https://doi.org/10.1002/acr.20579

INSEE. (2012). *Population by Age - Tables de l'Économie Française | Insee.* https://www.insee.fr/fr/statistiques/1372600?sommaire=1372680

INSEE. (2017). *4 million seniors would be in loss of autonomy in 2050-Insee Première-1767.* https://www.insee.fr/fr/statistiques/4196949

Jeannerod (2001). Neural Simulation of Action: A Unifying Mechanism for Motor Cognition. *NeuroImage*, *14*(1), S103-S109. https://doi.org/10.1006/nimg.2001.0832

Kharat, & Simonet. (2013). Diagnostic tools for acute confusional state. *Swiss Medical Journal.* https://www.revmed.ch/RMS/2013/RMS-370/Outils-diagnostiques-de-l-etat-confusionnel-aigu

Kubicki, A., & Mourey, F. (2015). Geriatric rehabilitation: A systems approach. *EMC Kinesitherapy-Physical Medicine-Rehabilitation*, *11*(4), 1-9.

Lundin-Olsson, L., Nyberg, L., & Gustafson, Y. (1997). "Stops walking when talking" as a predictor of falls in elderly people. *Lancet*, *349*(9052), 617. https://doi.org/10.1016/S0140-6736(97)24009-2

McKiernan, F. E. (2005). A simple gait-stabilizing device reduces outdoor falls and nonserious injurious falls in fall-prone older people during the winter. *Journal of the American Geriatrics Society*, *53*(6), 943-947. https://doi.org/10.1111/j.1532-5415.2005.53302.x

Menant, J. C., Schoene, D., Sarofim, M., & Lord, S. R. (2014). Single and dual task tests of gait speed are equivalent in the prediction of falls in older people: A systematic review and meta-analysis. *Ageing Research Reviews*, *16*, 83-104. https://doi.org/10.1016/j.arr.2014.06.001

Menant, J. C., Steele, J. R., Menz, H. B., Munro, B. J., & Lord, S. R. (2008). Optimizing footwear for older people at risk of falls. *Journal of Rehabilitation Research and Development*, *45*(8), 1167-1181.

Perera, S., Mody, S. H., Woodman, R. C., & Studenski, S. A. (2006). Meaningful change and responsiveness in common physical performance measures in older adults. *Journal of the American Geriatrics Society*, *54*(5), 743-749. https://doi.org/10.1111/j.1532-5415.2006.00701.x

Préfaut, & Ninot (2009). *Rehabilitation of the chronic respiratory patient.* https://www.elsevier-masson.fr/la-rehabilitation-du-malade-respiratoire-chronique-9782294048074.html

Professional Associations for Physical Activity, Sweden (2010). *Physical Activity in the Prevention and Treatment of Disease-Folkhälsomyndigheten.* http://www.folkhalsomyndigheten.se/publicerat-material/publikationsarkiv/p/physical-activity-in-the-prevention-and-treatment-of-disease/

Rulleau, T., Mauvieux, B., & Toussaint, L. (2015). Influence of circadian rhythms on the temporal features of motor imagery for older adult inpatients. *Archives of Physical Medicine and Rehabilitation*, *96*(7), 1229-1234. https://doi.org/10.1016/j.apmr.2015.02.015

Seichi, A., Hoshino, Y., Doi, T., Akai, M., Tobimatsu, Y., Kita, K., & Iwaya, T. (2014). Determination of the optimal cutoff time to use when screening elderly people for locomotive syndrome using the one-leg standing test (with eyes open). *Journal of Orthopaedic Science*, *19*(4), 620-626. https://doi.org/10.1007/s00776-014-0581-8

Temfemo, A., & Ahmaidi, S. (2018). Variation in the use of technical walking aids by older adults in EHPAD. *Clinical Neurophysiology*, *48*(6), 333. https://doi.org/10.1016/j.neucli.2018.10.060

Vass, C., Edwards, C., Smith, A., Sahota, O., & Drummond, A. (2015). What do patients wear on their feet? A service evaluation of footwear in elderly patients. *International Journal of Therapy and Rehabilitation*, *22*(5), 225-232. https://doi.org/10.12968/ijtr.2015.22.5.225

# List of annexes

***Appendix 1 -*** Short-Falls Efficacy Scale *International* Questionnaire

***Appendix 2 -*** Confusion Assessment *Method*

***Appendix 3 -*** *Medline* Anti-Slip Sock Instructions

## Appendix 1: Short-Falls Efficacy Scale International

<http://www.profane.eu.org/documents/SHORT_FES-I/Short_FES-I_Swiss_French.pdf>

## Appendix 2: Confusion Assessment

<https://www.revmed.ch/RMS/2013/RMS-370/Outils-diagnostiques-de-l-etat-confusionnel-aigu>

## Appendix 3: Medline Anti-Slip Socks Instructions
