# Supplementary material for: Evaluation of the impact of non-slip socks on the motor recovery of elderly people in acute care hospitals: Protocol for a randomized, controlled trial study
Source: PLoS One. 2023 May 1;18(5):e0283226. doi: 10.1371/journal.pone.0283226 (PMC10150981; doi:10.1371/journal.pone.0283226)
Supplement: S2 File — (DOCX) [file pone.0283226.s002.docx]

**Protocole ChARM**

**N° d’enregistrement :** n° 2021-A00349-32

**Ref :** CHD 20-0059

**«Evaluation de l’impact de Chaussettes Antidérapantes sur la Récupération Motrice des personnes âgées**

**Une Etude Pilote ouverte, monocentrique, randomisée, contrôlée »**

**Investigateur Coordonnateur :**

Thomas Rulleau,

Ingénieur de recherche - kinésithérapeute

Centre Hospitalier Départemental Vendée

Service de Court Séjour Gériatrique et Unité de Recherche Clinique

[thomas.rulleau@chd-vendee.fr](mailto:thomas.rulleau@chd-vendee.fr)

**Méthodologiste :**

**Lucie PLANCHE**

Centre Hospitalier Départemental Vendée

Unité de Recherche Clinique

[lucie.planche@chd-vendee.fr](mailto:lucie.planche@chd-vendee.fr)

**Promoteur :**

**Centre Hospitalier Départemental Vendée**Unité de Recherche Clinique
Boulevard Stéphane MOREAU
85 925 LA ROCHE SUR YON Cedex 09

Tel : 02 51 44 65 72
Fax : 02 51 44 65 85

Page de signature

**SIGNATURE DU PROMOTEUR**

| Le promoteur s’engage à réaliser cette étude selon toutes les dispositions législatives et réglementaires dont pourrait relever la recherche et selon le protocole. | | |
| --- | --- | --- |
| **Nom et fonction du représentant signataire :**  **Pour le promoteur et par délégation du Directeur Général, la Directrice des Affaires Médicales et de la Recherche** | **Date :** | **Signature :** |

**SIGNATURE DES INVESTIGATEURS/ Personne qualifiée**

| J'ai lu l’ensemble des pages du protocole de l’étude clinique dont le CHD Vendée est le promoteur. Je confirme qu'il contient toutes les informations nécessaires à la conduite de l’essai. Je m'engage à réaliser l’étude en respectant le protocole et les termes et conditions qui y sont définis. Je m'engage à réaliser l’étude en respectant :   - les principes de la “Déclaration d’Helsinki”, - les dispositions législatives et réglementaires du Code de la Santé Publique applicables aux RIPH de catégorie 2 ainsi que les textes d’application associés   Je m'engage également à ce que les investigateurs et les autres membres qualifiés de mon équipe aient accès à ce protocole ainsi qu’aux documents relatifs à la conduite de l’étude pour leur permettre de travailler dans le respect des dispositions figurant dans ces documents. | | | |
| --- | --- | --- | --- |
| **Investigateur coordonnateur** | **Nom :**  Thomas RULLEAU | **Date :** | **Signature :** |
| **Investigateur principal/ personne qualifiée** | **Nom et établissement :**  Thomas RULLEAU | **Date :** | **Signature :** |

LISTE DES ABREVIATIONS

| ANSM | Agence Nationale de Sécurité du Médicament et des produits de santé |
| --- | --- |
| ARC | Attaché de Recherche Clinique (moniteur) |
| BPC | Bonnes Pratiques Cliniques |
| CPP | Comité de Protection des Personnes |
| CNIL | Commission Nationale de l’Informatique et des Libertés |
| CRF | Case Report Form (cahier d’observation) |
| eCRF | Electronic Case Report Form (cahier d’observation électronique) |
| MR | Méthodologie de Référence CNIL |
| TEC | Technicien d'Etude Clinique |

Table des Matières

[Page de signature 2](#_Toc68769248)

[LISTE DES ABREVIATIONS 3](#_Toc68769249)

[Table des Matières 4](#_Toc68769250)

[INTRODUCTION 6](#_Toc68769251)

[1. Justification de l’etude 7](#_Toc68769252)

[1.1. Positionnement de la recherche 7](#_Toc68769253)

[1.2. Bénéfices et risques pour les personnes se prêtant à la recherche 9](#_Toc68769254)

[2. Objectifs et criteres de jugement 11](#_Toc68769255)

[2.1. Objectif et critère d’évaluation principal 11](#_Toc68769256)

[2.2. Objectifs et critères d’évaluation secondaires 11](#_Toc68769257)

[3. Population étudiée 12](#_Toc68769258)

[3.1. Description de la population 12](#_Toc68769259)

[3.2. Critères d'inclusion 12](#_Toc68769260)

[3.3. Critères de non-inclusion 12](#_Toc68769261)

[4. DESIGN ET Déroulement de l’étude 13](#_Toc68769262)

[4.1. Calendrier de l’étude 13](#_Toc68769263)

[4.2. Méthodologie générale de la recherche 16](#_Toc68769264)

[4.3. Schéma de l’étude 17](#_Toc68769265)

[4.4. Description et justification du schéma thérapeutique/ de la méthode étudiée 18](#_Toc68769266)

[4.5. Description de l’évaluation et des données recueillies 18](#_Toc68769267)

[4.6. Identification de toutes les données sources ne figurant pas dans le dossier médical 20](#_Toc68769268)

[4.7. Règles d'arrêt de la participation d’une personne 20](#_Toc68769269)

[4.8. INDEMNISATION 21](#_Toc68769270)

[5. EVALUATION DE LA SECURITE 22](#_Toc68769271)

[6. Data Management et statistiques 23](#_Toc68769272)

[6.1. Recueil et traitement des données de l’étude 23](#_Toc68769273)

[6.2. Statistiques 24](#_Toc68769274)

[7. Aspects administratifs et réglementaires 27](#_Toc68769275)

[7.1. Droit d'accès aux données et documents source 27](#_Toc68769276)

[7.2. Confidentialité des données 27](#_Toc68769277)

[7.3. Monitoring de l’étude 27](#_Toc68769278)

[7.4. Inspection / Audit 28](#_Toc68769279)

[7.5. délégation des taches 28](#_Toc68769280)

[7.6. déclaration aux autorités compétentes 28](#_Toc68769281)

[7.7. Amendements au protocole 28](#_Toc68769282)

[7.8. Données informatisées et soumission à la CNIL 29](#_Toc68769283)

[7.9. Information patient 29](#_Toc68769284)

[7.10. Financement et assurance 29](#_Toc68769285)

[7.11. Règles relatives à la publication 29](#_Toc68769286)

[7.12. Archivage des données sources 30](#_Toc68769287)

[8. Références bibliographiques 31](#_Toc68769288)

[Liste des annexes 35](#_Toc68769289)

[8.1. Annexe 1 : Short-Falls Efficacy Scale International 36](#_Toc68769290)

[8.2. Annexe 2 : Confusion Assessment 37](#_Toc68769291)

[8.3. Annexe 3 : Notice des Chaussettes antidérapantes medline 38](#_Toc68769292)

INTRODUCTION

Avec l’avancée en âge, de nombreux patients perdent leur autonomie. Cette perte d’autonomie peut être augmentée durant l’hospitalisation, par l’effet de la pathologie elle-même, ou par l’effet de l’hospitalisation. L’objectif des rééducateurs est de mettre en place des outils de préservation des capacités motrices des patients, par les séances de rééducation et/ ou avec du matériel adéquat. En particulier, ils s’assurent d’aide à la marche et d’un chaussage adapté à l’état clinique du patient.

Pourtant, les patients seniors arrivant en service de médecine n’ont pas toujours un chaussage adéquat. Les équipes les prenant en charge, dont les kinésithérapeutes, sont confrontées au choix de conserver le chaussage d’entrée ou de proposer aux patients de marcher pieds-nus. Le choix de conserver le chaussage habituel est le plus fréquent.

Depuis quelques temps, il est possible de proposer des chaussettes antidérapantes pour déambuler dans leur service. Cependant, il existe une controverse dans la littérature scientifique sur l’utilité de ces dispositifs. Il est en particulier impossible de transposer les résultats des études pour juger de l’intérêt de ces chaussettes chez le sujet senior.

L’objectif de cette étude est d’évaluer l’intérêt des chaussettes antidérapantes sur la récupération motrice.

# Justification de l’etude

## Positionnement de la recherche

### Rationnel

En 2012, les personnes âgées de 65 ans ou plus représentaient 17.1 % de la population (16 % en 2002), dont la moitié est représentée par celles âgées de 75 ans ou plus. On constate une hausse de 45% en 20 ans (INSEE, 2012). En 2015, en France, 2.5 millions de seniors sont en perte d’autonomie (INSEE, 2017). Parmi les seniors de 75 ans et plus, 8.8% vivent en institution (INSEE, 2017). La projection pour 2050 serait de 4 millions de seniors en perte d’autonomie, soit 16.4% des seniors (INSEE, 2017).

Fréquemment, lors d’une pathologie amenant une hospitalisation le vieillissement des personnes malades ou fragiles subissent un déconditionnement physique. Ce déconditionnement est un processus psychophysiologique conduisant à l’inactivité physique. Il est défini « comme un amplificateur de la vulnérabilité provoquant des situations de dépendance et une [qualité de vie](http://blogensante.fr/2013/09/02/definir-la-notion-de-qualite-de-vie/) altérée » (Préfaut & Ninot, 2009) **.** La masse, la force et la puissance musculaire diminuent avec l’âge, et à 80 ans les personnes âgées ont perdu la moitié de leur masse musculaire initiale (Professional Associations for Physical Activity, Sweden, 2010)

Figure 1: le cercle vicieux du déconditionnement d'après Hadjistavropoulos et al. (2011

D’après Hadjistavropoulos et ses collaborateurs (2011), un cercle vicieux déconditionnant existe chez le sujet senior. La chute entraine une peur de chuter, qui entraine une restriction d‘activité, qui entraine un déclin fonctionnel, qui entraine une diminution de la stabilité, qui entraine une majoration du risque de chute (**Erreur ! Source du renvoi introuvable.**).

Lors de leur arrivée dans les services de courts séjours, les patients peuvent être fatigués et/ou confus, selon les motifs de leur hospitalisation. A ce déconditionnement physique peut s’ajouter un déficit de stimulation qui sera un facteur aggravant, en particulier dans les premiers jours d’hospitalisation. Les kinésithérapeutes seront donc sollicités.

Les kinésithérapeutes veilleront à la bonne capacité motrice des sujets seniors. Marcher, effectuer des transferts, se mouvoir dans leur espace de vie ou encore vaquer aux activités de la vie quotidienne, est essentiel pour maintenir une qualité de vie dans le lieu de vie de son choix. (L’évaluation des capacités motrices sera développée ultérieurement). Une compétence importante des sujets âgés, outre l’intention de se mouvoir, est la planification motrice. La planification motrice est l’étape nécessaire pour construire la base du programme moteur. Parmi de nombreux autres mouvements possibles du fait de la redondance du système musculosquelettique, le sujet choisit un « mouvement type ». Cette planification motrice semble altérée chez certains patients âgés fragiles (Kubicki & Mourey, 2015). Dans cette mission de récupération des capacités motrices, le kinésithérapeute doit redonner dès que possible aux patients une marche sécurisante (auto-évaluation) et sécurisée (évaluation du kiné). Pour cela, et selon l’évaluation du kinésithérapeute, différentes aides techniques existent (Temfemo & Ahmaidi, 2018). Ces aides comprennent la canne simple, la canne tripode, les cannes anglaises, les déambulateurs avec ou sans roues etc. Le kinésithérapeute devra aussi faire attention aux chaussages.

En effet, un chaussage mal adapté peut-être vécu comme non sécurisé et/ou non sécurisant par l’équipe ou le patient. Luk et ses collaborateurs (2015) ont proposé une revue narrative sur la prévention de la chute chez les personnes âgées. Ils préconisent des interventions de prévention de la chute tels que l’exercice, des modifications de l’environnement, ou une révision de la médication. Ces auteurs proposent également de s’intéresser au chaussage des patients. Ils rapportent les propositions d’éviter les talons hauts et de mettre des chaussures antidérapantes sur la base d’autres travaux (McKiernan, 2005; Menant et al., 2008).

Le chaussage des sujets âgés en service de gériatrie a également été étudié par plusieurs équipes. Vass et ses collaborateurs (2015) ont examiné et décrit le type de chaussures portées par les patients âgés à l'hôpital. Ils montrent que de nombreux patients portent des chaussures dont la structure est insuffisante pour favoriser une stabilité ou une démarche optimale. Parmi les différents chaussages, ils retrouvent des chaussettes antidérapantes pour des sujets à domicile, en institution, ou en hôpital (Menant et al., 2008; Vass et al., 2015). Une revue narrative récente s’est intéressée à l’utilisation des chaussettes antidérapantes pour prévenir les chutes chez des patients âgés (Hartung & Lalonde, 2017). Dans leur conclusion, les auteurs ne recommandent pas le port des chaussettes pour les patients venus du domicile avec des limites sur les études retenues. Une limite importante est la validité externe des études, issues de la recherche fondamentale pour une part. L’autre part, issue de la recherche clinique ne s’intéresse pas spécifiquement à notre population de référence. De ce fait, il ne nous est pas possible de recommander ou non le port de chaussettes antidérapantes chez des patients seniors hospitalisés. Pourtant, la question se pose dans des conditions spécifiques que nous allons détailler.

En pratique clinique, dans les services de courts séjours de nombreux centres hospitaliers dont le CHD-Vendée, les patients arrivent des urgences sans chaussages adaptés ou sans chaussage du tout. La situation d’urgence les a conduits à l’hôpital sans leur laisser le temps de prendre des affaires. Lors des hospitalisations directes, une réévaluation du chaussage est aussi souvent nécessaire afin d’adapter le chaussage à la situation du patient. D’autres patients, en particulier ceux avec troubles cognitifs majeurs qui semblent le plus à risque, oublient régulièrement leurs chaussures la journée comme la nuit. Au final, l’autonomie semble s’en trouver altérée par l’absence de chaussures adaptées. Lors des prises en charge soignante (toilette, habillage etc.), les aides-soignants déplaceront le patient dans son fauteuil plutôt que de le faire marcher jusqu’au cabinet de toilette. En rééducation, le praticien peut choisir de ne pas prendre en charge un patient, ou de proposer une prise en charge partielle pour éviter la prise de risque lié à un chaussage inadapté. Le patient pourra se voir proposer de marcher seul en dehors des prises en charge rééducative.

### L’objectif de l’étude

Au final, nous voyons donc un intérêt clinique possible des chaussettes antidérapantes. La littérature scientifique présente des études avec des limites méthodologiques, mais surtout une population différente de celle qui nous intéresse. Il n’est donc actuellement pas possible de juger de l’intérêt spécifique de chaussettes antidérapantes chez une population âgée hospitalisée.

Evaluer l’intérêt de ces chaussettes pourrait permettre de les préconiser avec des données solides. Prévenir cette perte d’autonomie iatrogène entrainerait un impact positif sur les capacités motrices des patients, un retour vers le domicile plus rapide et plus facile, une baisse des coûts de santé.

Nous souhaiterions donc connaitre l’intérêt de porter des chaussettes antidérapantes dans la prise en charge de ces patients, et plus particulièrement sur l’objectif principal d’une rééducation en kinésithérapie gériatrique : la fonction motrice.

Nous pensons que le port de chaussettes antidérapantes améliore la récupération motrice des patients.

## Bénéfices et risques pour les personnes se prêtant à la recherche

### Bénéfices

#### Bénéfice individuel

Amélioration de la prise en charge motrice

Amélioration des pratiques avec utilisation de chaussettes antidérapantes

#### Bénéfice collectif

Amélioration de la prise en charge motrice de sujet senior permettant un coût moindre pour la société grâce à une durée de séjour plus courte,

Amélioration de la prise en charge motrice permettant une sortie plus rapide

### Contraintes et risques prévisibles

Contraintes :

Pour les deux groupes, la seule contrainte ajoutée par la recherche est la passation des tests et le remplissage du questionnaire spécifiques à la recherche.

Pour le groupe expérimental, la contrainte supplémentaire est le port des chaussettes antidérapantes.

Risques prévisibles :

Il n’existe pas de risque majoré par le port de chaussette antidérapante chez des patients séniors.

### Balance bénéfices / risques

Cette étude est qualifiée de recherche interventionnelle à risques et contraintes minimes telle que définie dans l’article L1121-1 du Code de la Santé Publique. Sur la base de l’ensemble des informations mentionnées précédemment, la balance bénéfices-risques de cette étude est évaluée favorablement.

# Objectifs et criteres de jugement

## Objectif et critère d’évaluation principal

### Objectif principal

Evaluer **l’impact des chaussettes antidérapantes sur la récupération motrice** des personnes âgées lors d’une hospitalisation

### Critère d’évaluation principal

Evolution **de la vitesse de marche à la vitesse de prédilection du patient sur 10m** entre J1 (début prise charge kiné) et J8. Deux tests seront réalisés à chaque évaluation, la moyenne des deux tests sera utilisée.

## Objectifs et critères d’évaluation secondaires

### Objectif(s) secondaire(s)

1. Evaluer l’impact des chaussettes antidérapantes sur les capacités motrices à court terme
2. Evaluer l’impact des chaussettes antidérapantes sur la planification motrice à court terme et durant l’hospitalisation
3. Evaluer l’impact du port des chaussettes sur les capacités à gérer la double tâche à court terme et durant l’hospitalisation
4. Evaluer l’évolution de la peur de la chute avec la mise des chaussettes à court terme et durant l’hospitalisation
5. Evaluer l’impact des chaussettes sur la présence de chute(s)
6. Durée de séjour en court séjour gériatrique ou médecine post-urgence

### Critère(s) d’évaluation secondaire(s)

1. Evolution de la vitesse de marche sur 10m entre J1 (début prise charge kiné) et J1’ (« après une marche de 3 minutes avec le chaussage attribué suite à la randomisation »),
2. Evolution de l’isochronie entre la vitesse de la marche réellement exécutée et la vitesse de la marche imaginée sur 10m à J1, J1’ et J8. La moyenne de deux tests de chaque modalité sera utilisée.
3. Evolution de l’isochronie entre la vitesse de marche et la vitesse de marche en double tâche sur 10m à J1,J1’ et J8 (Beauchet et al., 2010; Menant et al., 2014). La moyenne de deux tests de chaque modalité sera utilisée.
4. Score total > 10 au questionnaire Short -Falls Efficacy Scale International à J1, J1’ et J8
5. Présence de chute(s) et ses conséquences durant l’hospitalisation
6. Durée de séjour calculée entre le début de l’hospitalisation et la fin de l’hospitalisation

# Population étudiée

## Description de la population

Cette étude s’adresse aux patients admis au CHD Vendée en Médecine Post-Urgence et Court Séjour Gériatrique et nécessitant une prise en charge kinésithérapique.

## Critères d'inclusion

- Patient en Court Séjour Gériatrique ou Médecine Post-Urgence,
- Patient de 75 ans et plus,
- Patient nécessitant de la kinésithérapie,
- Patient avec une durée de prise en charge en kinésithérapie sur 7 jours minimum
- Patient arrivé avec des chaussures inadaptées (évaluation à l’appréciation du clinicien : pas de contrefort arrière, taille inadaptée etc.) ou sans chaussure,
- Patient apte à la marche, au moins 10 m avec ou sans aide technique,
- Patient ayant donné son consentement oral
- Patient bénéficiant d’une couverture sociale.

## Critères de non-inclusion

- Incapacité à comprendre ou réaliser les tests cliniques spécifiques à l’étude
- Patients avec des antécédents de démence évalués antérieurement à son hospitalisation, avec un score au MMSE ≤ 22/30
- Patient non voyant
- Patient sous tutelle ou curatelle
- Patient participant à un protocole de recherche clinique de type interventionnel susceptible de modifier les évaluations du présent protocole.
- Patient avec impossibilité de mettre des chaussettes ou de marcher pieds nus (besoin de bas ou chaussettes de contention, plaie, œdème trop important, autre)

L’investigateur/ la personne qualifiée, ou une personne désignée par celui-ci, devra tenir à jour une liste des patients non inclus tout au long de l’étude. Cette liste devra contenir l’ensemble des patients issus de la population mais ne répondant pas aux critères d’éligibilité. Il devra également y indiquer, pour chaque patient, le motif de non-inclusion dans l’étude.

# DESIGN ET Déroulement de l’étude

## Calendrier de l’étude

### Inclusion et Randomisation

Les patients arrivent dans le service et sont évalués par le médecin qui prescrit, si nécessaire, de la kinésithérapie.

Le kinésithérapeute prend connaissance de la prescription lors du premier jour ouvré suivant. Le kinésithérapeute effectue en pratique courante une analyse du chaussage du patient. Si ce chaussage n’est pas adapté ou si le patient est pieds nus, le kinésithérapeute vérifie les critères d’éligibilité du patient. Si le patient respecte ces critères, il est informé de l’étude. Si le patient donne son consentement oral, le kinésithérapeute effectuera la **randomisation.**

**Bras de randomisation :**

- **Bras contrôle : prise en charge habituelle avec les pieds nus.**
- **Bras expérimental : prise en charge spécifique avec des chaussettes antidérapantes.**

### Baseline : Tests J1

Le kinésithérapeute évalue le patient lors de son bilan-diagnostic de première séance et réalise l’échelle *Confusion Assesment Method* si elle n’a pas été effectuée dans la pratique courante.

Une Evaluation Visuelle Analogique de la fatigue sera réalisée avant les tests cliniques.

Dans le cadre de la recherche, les tests **J1** seront réalisés pieds nus :

1. Vitesse de marche du patient sur 10 m (2 fois)
2. Vitesse de marche en situation de double tâche sur 10 m (2 fois)

Deux autre évaluations moins courantes mais validées dans cette population seront réalisées.

1. Vitesse de marche imaginée sur 10 m (2 fois)

La moyenne de chacun des deux tests sera retenue pour les items 1 à 4.

L’ensemble de ces tests représentent une durée pour le patient d’environ 10 minutes.

1. Questionnaire short-Falls Efficacy Scale International

En fonction du bras de randomisation, le kinésithérapeute mettra des chaussettes antidérapantes aux patients du bras expérimental et laissera les patients du bras contrôle pieds nus. Il incitera ensuite le patient à marcher pendant 3 minutes.

### Suivi J1’

Après ces 3 minutes de marche effectuées avec ou sans les chaussettes en fonction du bras de randomisation et afin d’évaluer l’effet immédiat du port des chaussettes antidérapantes sur les capacités motrices, le patient effectuera les mêmes tests qu’à J1 dans le même ordre.

Une Evaluation Visuelle Analogique de la fatigue sera réalisée avant les tests cliniques suivants :

1. Vitesse de marche du patient sur 10 m (2 fois)
2. Vitesse de marche en situation de double tâche sur 10 m (2 fois)
3. Vitesse de marche imaginée sur 10 m (2 fois)

Les tests seront effectués par le patient avec les pieds nus (bras contrôle) ou avec les chaussettes antidérapantes (bras expérimental).

### Procédure de prise en charge du patient durant le séjour

Suite à cette seconde évaluation et pendant toute la durée d’hospitalisation, les chaussettes seront laissées aux patients dans le bras expérimental, et les patients du bras contrôle resteront pieds nus. Un affichage sera prévu dans les chambres des patients afin d’informer les équipes soignantes pour que le bras de randomisation soit respecté.

Entre J1’ et J8, le patient sera suivi en rééducation selon le plan préétabli d’après les conclusions du bilan-diagnostic masso-kinésithérapique (BDMK). Le patient bénéficiera de 3 séances, la première incluant le bilan à J1.

### Suivi J8

A J8, afin d’évaluer l’effet des chaussettes sur la préservation/ récupération motrice, le patient sera réévalué dans les mêmes conditions qu’à J1 soit, pieds nus, par les mêmes tests qu’à J1 et dans le même ordre.

Une Evaluation Visuelle Analogique de la fatigue sera réalisée avant les tests cliniques suivants :

1. Vitesse de marche du patient sur 10 m (2 fois)
2. Vitesse de marche en situation de double tâche sur 10 m (2 fois)
3. Vitesse de marche imaginée sur 10 m (2 fois)
4. Questionnaire short-Falls Efficacy Scale International

**Calendrier de l’étude**

| **Actions** | J1 | J1’ (après 3 minutes de marche libre) | J8 | Sortie d’hospitalisation |
| --- | --- | --- | --- | --- |
| Information du patient | X |  |  |  |
| Recueil du Consentement éclairé | X |  |  |  |
| Vérification des critères d’inclusion et de non-inclusion | X |  |  |  |
| Randomisation | X |  |  |  |
| *Confusion Assessment Method* | X |  |  |  |
| EVA fatigue | X | X | X |  |
| Antécédents (chute, MMSE etc.) | X |  |  |  |
| Type d’aide technique | X | X | X |  |
| Examen clinique | X |  | X |  |
| Tests :   - Vitesse de marche à vitesse de prédilection du patient sur 10m (x2), - Vitesse de marche en double tâche sur 10m (x2), - Vitesse de marche imaginée sur 10m (x2), | X | X | X |  |
| Questionnaire Short-FESI | X |  | X |  |
| Durée de séjour en court séjour gériatrique ou médecine post-urgence |  |  |  | X |

.

## Méthodologie générale de la recherche

La recherche présente les caractéristiques suivantes :

- Etude ouverte,

- Monocentrique (CHD Vendée)

- Contrôlée,

- De supériorité,

- Randomisée

Durée d’inclusion : 18 mois

Durée de participation : 7 jours

Durée de la recherche : 19 mois maximum

Nombre de patients à randomiser : 50

## Schéma de l’étude

Indication et prescription

Critères d’éligibilité satisfaits

Consentement et inclusion

Randomisation

Evaluation J1 :

Evaluation pieds nus

1° bras :

Chaussettes antidérapantes

2° bras :

Pieds nus

Evaluation J1’ à 3 minutes

Évaluation avec chaussettes

antidérapantes

Evaluation J1’ à 3 minutes

Évaluation pieds nus

Rééducation selon le bilan-diagnostic

masso-kinésithérapique

Rééducation selon le bilan-diagnostic

masso-kinésithérapique

Evaluation J8

Pieds nus

## Description et justification du schéma thérapeutique/ de la méthode étudiée

L’objectif de l’étude est d’évaluer l’effet du port de chaussettes antidérapantes sur la récupération motrice. Nous avons donc choisi un design où nous pourrons évaluer l’évolution des capacités motrices entre un groupe contrôle et le groupe expérimental porteur de chaussettes.

Les chaussettes antidérapantes utilisées dans cette étude (annexe 3) seront celles utilisées en pratique habituelle dans notre service de soin.

Ces chaussettes de la marque Medline possèdent des bandes à picots sur les 2 faces externes permettant une adhérence en continue, même si la chaussette tourne. Elles disposent d’un intérieur en tissu éponge absorbant permettant ainsi de conserver les pieds au sec. Elles sont sans latex évitant des allergies.

Les chaussettes seront changées chaque jour pour garder une bonne hygiène des pieds. Le changement sera fait par le personnel médical au lit du patient.

## Description de l’évaluation et des données recueillies

### La vitesse de marche

La vitesse de marche est un test courant en Kinésithérapie utilisé pour évaluer la fonction motrice. Il est simple à utiliser même pour les patients avec des troubles cognitifs et confusionnels. La vitesse de marche des patients âgés de plus de 80 ans est de 0.943m/secondes ± 0.091 (Bohannon & Williams Andrews, 2011; Menant et al., 2014)

Les meilleures estimations initiales de petits changements significatifs sont proches de 0,05 m/s pour la vitesse de marche. (Perera et al., 2006)

Les changements substantiels sont de l’ordre de 0.10 m/s (Perera et al., 2006).

Il existe plusieurs modalités de passation du test, à vitesse de marche habituelle ou à vitesse de marche rapide. Dans le cadre de ce protocole, nous avons choisi la modalité marche à vitesse de prédilection du patient (Bohannon & Williams Andrews, 2011).

Des marquages au sol ou des plots permettront de donner un repère visuel au patient. Le patient est debout face au parcours de 10m, avec les repères visuels qui lui sont rappelés.

Le test de la vitesse de marche sera effectué 2 fois. Les consignes sont :

« À mon top de départ, vous devrez marcher à votre vitesse habituelle pour dépasser le repère».

« Êtes-vous prêt ? »

### La vitesse de marche en double tâche

La vitesse de marche en double tâche donne une indication de la réserve cognitive disponible (Lundin-Olsson et al., 1997). Un arrêt de la marche ou une diminution de la vitesse de marche de plus de 30% est un facteur prédictif de chute (Beauchet & Berrut, 2006).

Des marquages au sol ou des plots permettront de donner un repère visuel. Le patient est debout face au parcours de 10m, avec les repères visuels qui lui sont rappelés.

Le test de la vitesse de marche sera effectué 2 fois. Les consignes sont :

« À mon top de départ, vous devrez marcher à votre vitesse habituelle pour dépasser le repère/plot au sol. Pendant cette marche, vous devrez me citer le plus de noms d’animaux possible ».

« Êtes-vous prêt ? »

### La vitesse de marche imaginée

Le mouvement humain est séparable en 2 phases, une phase de programmation (anticipation) et une phase d’exécution (Jeannerod, 2001). La concordance entre la planification et l’exécution du mouvement peut-être évalué de manière fiable grâce à un test simple évaluant la concordance temporelle entre une pratique imaginée et une pratique physique (Guillot et al., 2012). Cette concordance se traduit par un Indice d’Isochronie. Adapté à la vitesse de marche imaginée, c’est un test simple visant à évaluer les capacités de prédiction motrice (Rulleau et al., 2015). De façon intéressante, la diminution de l’isochronie est corrélée à la diminution de la vitesse de marche lors de la double tâche (Bridenbaugh et al., 2013)). Il pourrait ainsi marquer un risque de chute par mauvaise anticipation des capacités motrices réelles.

Des marquages au sol ou des plots permettront de donner un repère visuel. Le patient est debout face au parcours de 10m, avec les repères visuels qui lui sont rappelés.

Le test de la vitesse de marche imaginée sera effectué 2 fois. Les consignes sont

« À mon top de départ, vous devrez, en restant à votre place, vous imaginer marcher à votre vitesse habituelle pour dépasser le repère/plot au sol. Quand vous imaginerez le dépasser vous me direz « stop ».

« Êtes-vous prêt ? »

### La peur de la chute (Annexe 2)

La short-Falls Efficacy Scale International (Short-FES-I) court est un auto-questionnaire remplie par les patients et composée de 7 items avec 4 réponses possibles. Elle présente une excellente cohérence interne (coefficient alpha de Cronbach = 0,96 et 0,92) et une fiabilité test-retest (ICC = 0,96 et 0,83). La validité conceptuelle convergente de la Short-FES-I a été confirmée pour : les chutes antérieures; les symptômes de dépression; l'incapacité générale; la faible qualité de vie; et la déficience physi-calorique (Dewan & MacDermid, 2014).

Un seuil pour une peur de la chute a été défini comme > 10 sur l'échelle Short-FES-I (Dewan & MacDermid, 2014).

### La présence d’une chute

La survenue d’une chute pendant l’hospitalisation, l’heure de la chute et ses conséquences, le nombre total de chutes pendant la durée d’hospitalisation, sera répertorié.

Seront récoltées aussi les chutes et dates de chutes précédent l’hospitalisation de moins de 6mois.

### Confusion Assesment Method (annexe 3)

Cet outil est destiné à être utilisé par un personnel soignant, qui recherche 4 groupes de signes cliniques (Haute Autorité de Santé, 2009; Kharat & Simonet, 2013) :

1. Début soudain et fluctuation des symptômes ;
2. Inattention ;
3. Désorganisation de la pensée ;
4. Troubles de la vigilance.

Le diagnostic requiert la présence de 3 des 4 critères. Les critères 1 et 2 sont toujours requis, accompagnés du 3 ou 4 (Kharat et Simonet, 2013 ; Annexe 3).

### Echelle visuelle analogique de la fatigue

L’échelle visuelle analogique est couramment utilisée dans l’évaluation de la douleur (Hawker et al., 2011). En complément, d’autres auteurs proposent sont utilisation dans l’évaluation de la fatigue sous la même modalité (Hewlett et al., 2011).

La réglette est présentée au patient sous ces termes :

« ceci est une réglette représentant votre fatigue, à gauche pas de fatigue du tout, à droite, la fatigue maximale que vous puissiez imaginer. Je vais vous demander de déplacer le curseur à votre niveau de fatigue actuelle ».

L’opérateur côte la fatigue de 0 à 100.

### Durée de séjour

La durée de séjour en jour et heure en court séjour gériatrique ou médecine post-urgence calculée entre le début de l’hospitalisation et la fin de l’hospitalisation sera répertoriée.

## Identification de toutes les données sources ne figurant pas dans le dossier médical

1. Echelle Confusion Assesment Method à J1
2. Echelle Visuelle Analogique de la fatigue à J1, J1’ et J8
3. Type d’aide(s) technique à J1, J1’, J8
4. Durée de la marche sur 10 m (2 fois) réalisée à J1, J1’ et J8
5. Durée de la marche en double tâche sur 10 m (2 fois) réalisée à J1, J1’ et J8
6. Durée de la marche imaginée sur 10 m (2 fois) réalisée à J1, J1’ et J8
7. Questionnaire short-Falls Efficacy Scale International réalisé à J1, J1’ et J8

## Règles d'arrêt de la participation d’une personne

### Critères d’arrêt prématuré de la participation d'une personne à la recherche

La participation d’une personne peut être arrêtée prématurément pour les raisons suivantes :

- Retrait du consentement par le patient

Les patients pourront retirer leur consentement et demander à sortir de l’étude à n’importe quel moment et quelle qu’en soit la raison.

- Décès.

### Modalité de suivi et calendrier de recueil des données

Les patients qui seront sortis de l’étude, ne poursuivront pas le calendrier de l’étude.

En cas de retrait de consentement du patient, et sans préjudice pour le droit du patient ; les données déjà recueillies feront l’objet d’une analyse conformément aux textes règlementaires.

En revanche, aucun autre examen prévu spécifiquement par le protocole ne sera réalisé et aucune donnée ne sera recueillie.

La sortie d'étude d'un patient ne changera en rien sa prise en charge habituelle par rapport à sa pathologie.

### Critères d’arrêt d'une partie ou de la totalité de la recherche (hors considérations biostatistiques)

Une partie ou la totalité de l'étude peut être arrêtée définitivement ou temporairement sur décision de l'ANSM, du CPP et/ou du Promoteur de l'étude.

Dans tous les cas :

- Une confirmation écrite sera envoyée à l'investigateur coordonnateur de l'étude (précisant les raisons d'arrêt prématuré)*,*

-Tous les patients de l'étude seront informés.

### Modalité de prise en charge des patients à la fin de la recherche

Les patients seront pris en charge selon les pratiques habituelles du service.

## INDEMNISATION

Aucune indemnisation n’est prévue pour la participation à cette étude.

# EVALUATION DE LA SECURITE

L’étude correspondant à une RIPH (Recherche Impliquant la Personne Humaine) de catégorie 2, les dispositions applicables en matière de vigilance sont celles mises en place dans la pratique des soins et l’utilisation des produits associés à l’étude conformément à l’article L1123-10 du Code de la santé publique. L’investigateur sera donc responsable de déclarer la survenue de tout éventuel évènement indésirable selon les procédures applicables au CHD Vendée.

# Data Management et statistiques

## Recueil et traitement des données de l’étude

### Recueil des données

Un cahier d’observation (eCRF) sera créé par patient. Toutes les informations requises par le protocole doivent être fournies dans l’eCRF. Il doit comprendre les données nécessaires pour confirmer le respect du protocole et toutes les données nécessaires aux analyses statistiques ; il doit permettre de déceler les écarts majeurs au protocole.

La/les personne(s) responsable(s) du remplissage des eCRF (investigateur, ARC…) devra /devront être définie(s) et est/sont identifiée(s) dans le formulaire de délégations des tâches (conservé dans le classeur investigateur).

En fin d’étude, l’investigateur signera les eCRF afin d’attester la conformité des données recueillies.

### Codage des données

En signant ce protocole l’investigateur principal / la personne qualifiée et l’ensemble des membres de son équipe s’engagent à maintenir confidentielles les identités des patients ou patientes qui ont participé à l’étude.

La transmission des données d’une personne à des fins de recherche ne sera dès lors possible que sous réserve de l’apposition d’un système de codage ; la présentation des résultats de la recherche exclura toute identification directe ou indirecte.

L’identification des patients se fera selon l’ordre d’inclusion des patients par un numéro attribué automatiquement par le logiciel Ennov Clinical (eCRF) puis complété par les initiales des patients (1ère lettre du prénom + 1ère lettre du nom).

Ce code sera la seule information qui figurera sur l’eCRF et qui permettra de rattacher à posteriori l’eCRF au patient.

L’investigateur / la personne qualifiée est également tenu de coder les données patients sur tous les documents qu’il pourrait avoir en sa possession qui seraient joints à l’eCRF.

Une table de correspondance sera mise en place au sein du centre participant. Cette table sera conservée dans un endroit sécurisé par l’investigateur principal / la personne qualifiée du centre et contiendra le code patient et ses données nominatives afin de pouvoir remonter au dossier patient en cas de données manquantes ou erronées. Aucune donnée clinique ne sera recueillie dans ces tables de correspondances.

### Traitement des données

La collecte des données cliniques reposera sur la mise en place d'une base de données et la création de masques de saisie à l’image du cahier d'observation en conformité avec le protocole et les réglementations actuellement en vigueur.

## Statistiques

Logiciel

Les analyses seront réalisées sous le logiciel R version 3.5.1

### Description des méthodes statistiques prévues, y compris du calendrier des analyses intermédiaires prévues

L’ensemble des variables sera décrit globalement et par groupe. La description comprendra les effectifs et pourcentages des modalités pour les variables qualitatives et les minimum, maximum, moyenne, écart-type et médiane pour les variables quantitatives.

L’ensemble des critères vitesse de marche, isochronie entre la vitesse de marche imaginée et exécutée , isochronie entre vitesse de marche normale et marche en double tâche seront comparés à l’aide de modèles linéaires tenant compte de la valeur baseline J1.

Les conditions d’évaluation entre J1’ et J8 étant différentes, les modèles évaluant l’évolution à J1’ et à J8 seront indépendants.

La peur de la chute sera évaluée à partir du questionnaire Falls Efficacy Scale International. La peur de la chute est définie avec un cut-off égal 10 au score de ce questionnaire.

Le nombre de patients avec son pourcentage ayant une peur de la chute sera présenté et comparé à J1’ et à J8 à l’aide d’un test du Khi2.

La durée de séjour sera comparée à l’aide d’un test de Student.

La présence de chutes ainsi que leur conséquence seront décrites au cours du séjour en hospitalisation.

### Justification statistique du nombre d’inclusions

La vitesse de marche est un test courant en Kinésithérapie utilisé pour évaluer la fonction motrice. Il est simple à utiliser même pour les patients avec des troubles cognitifs et confusionnels. La vitesse de marche des patients âgés de plus de 80 ans est de 0.943m/secondes ± 0.091 (Bohannon & Williams Andrews, 2011; Menant et al., 2014)

D’après Perera et al., amélioration de la vitesse de marche de 0.10m/s est considérée comme cliniquement pertinente (Perera et al., 2006).

Dans le cadre de cette étude, nous retenons donc une différence de 0.10m/s à mettre en évidence entre les 2 groupes et un écart-type de la différence fixé à 0.10.

D’après ces hypothèses et pour un risque alpha fixé à 5% et une puissance à 90%, 46 patients au total sont nécessaires. Afin de garantir la puissance de l’étude, 50 patients seront randomisés.

### Degré de signification statistique prévu

Le risque alpha est fixé à 5%

### Critères statistiques d'arrêt de la recherche

NA

### Méthode de prise en compte des données manquantes, inutilisées ou non valides

Toutes les données manquantes ainsi que leur raison seront décrites dans chacun des groupes.

Pour le critère principal, si des données manquantes sont présentes à J8 elles seront imputées par la valeur baseline du patient (J1).

Si pour ce critère, plus de 10% de données manquantes sont observées, une analyse de sensibilité sur la méthode d’imputation sera réalisée : une méthode d’imputation multiple sera appliquée.

### Gestion des modifications apportées au plan d'analyse de la stratégie initiale

NA

### Choix des personnes à inclure dans les analyses

L’analyse principale sera réalisée sur la population en Intention de Traiter (ITT), c’est-à-dire sur l’ensemble des patients randomisés.

Une analyse complémentaire sera réalisée sur la population en Per Protocol (PP) incluant les patients randomisés pour lesquels aucune déviation majeure au protocole n’aura été relevée.

Une réunion de revue des données sera organisée afin de revoir et définir le critère majeur ou non de chacune des déviations.

### Randomisation

La randomisation ne sera pas stratifiée

Elle sera effectuée selon un ratio 1 :1 et sera réalisée par blocs.

La randomisation sera réalisée sous Ennov Clinical par la connexion au site internet : <https://nantes-lrsy.hugo-online.fr/EnnovClinical/>. La connexion s’effectuera grâce à un login, un mot de passe et un numéro d’étude, délivrés par le data-manager de l’Unité de Rechercher du CHD de La Roche sur Yon. Les informations suivantes devront être renseignées :

- Première initiale du nom,

- Première initiale du prénom,

- Mois et Année de naissance,

- Respect des critères d’inclusion et de non inclusion (oui/non),

La randomisation sera effectuée par le kinésithérapeute ou toute autre personne autorisée après confirmation de la possibilité de l’inclusion du patient dans l’étude et recueil de son consentement oral. La randomisation sera réalisée avant d’effectuer les évaluations J1. Le numéro d’inclusion sera attribué automatiquement lors de la randomisation. Une confirmation par mail sera envoyée à la personne ayant effectuée la randomisation ainsi qu’à toutes les personnes concernées.

La liste de randomisation sera réalisée par l’équipe biométrie de l’Unité de Recherche du CHD de La Roche sur Yon. Un guide explicatif de la randomisation sera disponible en ligne sous Ennov Clinical.

# Aspects administratifs et réglementaires

## Droit d'accès aux données et documents source

Les investigateurs mettront à disposition des personnes chargées du suivi, du contrôle de qualité ou de l'audit de la recherche, les documents et données individuelles strictement nécessaires à ce contrôle, conformément aux dispositions législatives et réglementaires en vigueur (articles L.1121-3 et R.5121-13 du code de la santé publique).

## Confidentialité des données

Les personnes ayant un accès direct prendront toutes les précautions nécessaires en vue d'assurer la confidentialité des informations relatives aux personnes qui s'y prêtent et notamment en ce qui concerne leur identité ainsi qu’aux résultats obtenus.

Ces personnes, au même titre que les investigateurs eux-mêmes, sont soumises au secret professionnel (selon les conditions définies par les articles 226-13 et 226-14 du code pénal).

Pendant la recherche ou à son issue, les données recueillies sur les personnes qui s’y prêtent et transmises par les intervenants seront pseudonymisées.

Elles ne doivent en aucun cas faire apparaître en clair les noms des personnes concernées ni leur adresse.

Seule la première lettre du nom de la personne et la première lettre de son prénom ainsi que son année de naissance seront enregistrées, accompagnées d’un numéro codé propre à l’étude indiquant l’ordre d’inclusion des patients*.*

## Monitoring de l’étude

Le monitoring sera assuré par le Département promotion de la Direction de la recherche. Un Attaché de Recherche Clinique (ARC) se rendra régulièrement sur chaque site afin de procéder au contrôle qualité des données rapportées dans les cahiers d’observations.

Le plan de monitoring est défini et adapté au niveau de risque estimé pour le patient se prêtant à la recherche. Il sera suivi de la manière suivante :

Risque A : risque prévisible faible ou négligeable

Les visites de monitoring sur site seront organisées après rendez-vous avec l’investigateur/ la personne qualifiée. Les ARC devront pouvoir consulter :

- les cahiers de recueil de données des patients inclus,

- les dossiers médicaux et infirmiers des patients,

- le classeur investigateur.

## Inspection / Audit

Dans le cadre de la présente étude, une inspection ou un audit pourra avoir lieu. Le promoteur et/ou le centre participant doivent pouvoir donner l’accès aux données aux inspecteurs ou auditeurs.

## délégation des taches

L’investigateur principal du lieu de recherche établit et tient à jour le formulaire de délégations des tâches qui précise les tâches respectives qu’il délègue aux membres de son équipe dans le cadre de l’étude, selon leur compétence.

Chaque collaborateur de l'investigateur établit un curriculum vitae (CV) daté et signé et tenu à jour.

L’investigateur veille à ce que les collaborateurs à qui il délègue des tâches dans le cadre de l’étude ont la compétence appropriée pour ces tâches. Il reste responsable de la conduite de la recherche sur le lieu.

## déclaration aux autorités compétentes

Le promoteur s’engage à soumettre le projet d’étude à l’autorisation préalable d’un Comité de Protection des Personnes (CPP). Les informations communiquées portent d’une part sur les modalités et la nature de la recherche et d’autre part, sur les garanties prévues pour les patients participant à cette étude. Le promoteur soumet à l’avis du CPP le curriculum vitae de l’investigateur principal du lieu de recherche.

.

Le présent protocole fera également l’objet d’une information auprès de l’ANSM.

## Amendements au protocole

Les demandes de modifications substantielles seront adressées par le promoteur pour avis au CPP concerné conformément à la loi en vigueur et ses arrêtés d'application.

Le protocole modifié devra faire l’objet d’une version actualisée datée et signée.

Les lettres d’information et le formulaire de recueil consentement oral du patient devront faire l’objet de modification si nécessaire.

## Données informatisées et soumission à la CNIL

Les données recueillies dans le cadre de cette étude le sont dans un but de recherche scientifique, à des fins d’intérêt public.

Cette étude entre dans le cadre de la « Méthodologie de Référence » MR-001 enregistrée, pour le CHD Vendée, sous le n°2060482 v 0 pour les raisons suivantes :

- Recueil de données de santé à des fins de recherche

- Obtention de l’avis d’un CPP pour débuter la recherche

- Utilisation de données pseudonymisées

- Information individuelle des personnes concernées

- Accès aux données uniquement par les professionnels (de santé et du promoteur) impliqués dans l’étude.

Le fait que cette étude entre dans le cadre de la MR001 ainsi que les raisons seront notifiées dans le registre des traitements du promoteur.

## Information patient

### Consentement éclairé oral

L’investigateur/ la personne qualifiée s’engage à obtenir le consentement libre, éclairé et exprès de la personne, recueilli par oral, après lui avoir délivré l’information sur le protocole. Il lui remettra un exemplaire de la note d’information. La personne ne pourra être incluse dans l’étude qu’après avoir pris connaissance de la note d’information et donné son consentement oral après avoir disposé, si nécessaire, d’un temps de réflexion.

L’information du patient et son accord pour participer à la recherche devront être notées dans son dossier médical.

Une traçabilité du recueil du consentement oral exprès du patient sera conservée dans les documents de l’étude.

## Financement et assurance

Le promoteur assure le financement de l’étude et souscrit une police d’assurance garantissant les conséquences pécuniaires de sa responsabilité civile, conformément à la réglementation.

## Règles relatives à la publication

L’étude sera enregistrée sur le site web en libre accès Clinical trial avant l'inclusion du 1^er^ patient dans cette étude.

L'étude ne pourra faire l'objet d'aucun commentaire écrit ou oral sans l'accord du promoteur ; l'ensemble des informations communiquées ou obtenues pendant la réalisation de l'étude appartenant de plein droit au CHD Vendée qui pourra librement en disposer.

Toutes les informations résultant de cette étude sont considérées comme confidentielles, au moins jusqu'à ce que l'analyse appropriée et le contrôle par le promoteur, le coordonnateur et le statisticien de l'étude soient achevés.

Les communications et rapports scientifiques correspondant à cette étude seront réalisés sous la responsabilité du coordonnateur de l'étude.

Le coordonnateur de l'étude sera le signataire principal de la communication et le rédacteur des documents, il figurera nécessairement aux rangs du premier ou dernier auteur. Il peut éventuellement déléguer cette tâche à une autre personne.

L’investigateur coordonnateur établit la liste des auteurs. Les investigateurs seront cités au prorata du nombre de patients recrutés. Le statisticien de l'étude sera également cité.

De même, les publications des résultats annexes comporteront le nom de la personne ayant réalisé le travail annexe ainsi que les noms de toutes les autres personnes concernées par ce travail annexe.

Toutes publications, abstracts ou présentations comprenant les résultats de l'étude doivent être soumis pour approbation au promoteur (CHD Vendée).

Les règles de publications suivront les recommandations internationales (N Engl J Med, 1997; 336 :309-315). .

## Archivage des données sources

L’investigateur / la personne qualifiée doit conserver toutes les informations relatives à l’étude pour au moins 15 ans après la fin de l’étude.

A la fin de l’étude, l’investigateur / la personne qualifiée recevra une copie des données de chaque patient envoyée par le promoteur.

Aucun déplacement ou destruction ne pourra être effectué sans l’accord du Promoteur. Au terme des 15 ans, le promoteur sera consulté pour destruction. Toutes les données, tous les documents et rapports pourront faire l’objet d’audit ou d’inspection.

# Références bibliographiques

Beauchet, O., Annweiler, C., Assal, F., Bridenbaugh, S., Herrmann, F. R., Kressig, R. W., & Allali, G. (2010). Imagined Timed Up & Go test: A new tool to assess higher-level gait and balance disorders in older adults? *Journal of the Neurological Sciences*, *294*(1–2), 102–106. https://doi.org/10.1016/j.jns.2010.03.021

Beauchet, O., & Berrut, G. (2006). [Gait and dual-task: Definition, interest, and perspectives in the elderly]. *Psychologie & neuropsychiatrie du vieillissement*, *4*(3), 215–225.

*Bilan social 2015 | Publication ATIH*. (2015). https://www.atih.sante.fr/bilan-social-2015

Bohannon, R. W., & Williams Andrews, A. (2011). Normal walking speed: A descriptive meta-analysis. *Physiotherapy*, *97*(3), 182–189. https://doi.org/10.1016/j.physio.2010.12.004

Bridenbaugh, Beauchet, Annweiler, Allali, Herrmann, F., & Kressig, R. W. (2013). Association between dual task-related decrease in walking speed and real versus imagined Timed Up and Go test performance. *Aging Clinical and Experimental Research*, *25*(3), 283–289. https://doi.org/10.1007/s40520-013-0046-5

Dewan, N., & MacDermid, J. C. (2014). Fall Efficacy Scale-International (FES-I). *Journal of Physiotherapy*, *60*(1), 60. https://doi.org/10.1016/j.jphys.2013.12.014

Guillot, Hoyek, Louis, & Collet. (2012). *Understanding the timing of motor imagery: Recent findings and future directions*. *5*(1), 3–22.

Hadjistavropoulos, T., Delbaere, K., & Fitzgerald, T. D. (2011). Reconceptualizing the role of fear of falling and balance confidence in fall risk. *Journal of Aging and Health*, *23*(1), 3–23. https://doi.org/10.1177/0898264310378039

Hartung, B., & Lalonde, M. (2017). The use of non-slip socks to prevent falls among hospitalized older adults: A literature review. *Geriatric Nursing (New York, N.Y.)*, *38*(5), 412–416. https://doi.org/10.1016/j.gerinurse.2017.02.002

Haute Autorité de Santé. (2009). *Confusion aiguë chez la personne âgée: Prise en charge initiale de l’agitation*. Haute Autorité de Santé. https://www.has-sante.fr/jcms/c_819557/fr/confusion-aigue-chez-la-personne-agee-prise-en-charge-initiale-de-l-agitation

Hawker, G. A., Mian, S., Kendzerska, T., & French, M. (2011). Measures of adult pain: Visual Analog Scale for Pain (VAS Pain), Numeric Rating Scale for Pain (NRS Pain), McGill Pain Questionnaire (MPQ), Short-Form McGill Pain Questionnaire (SF-MPQ), Chronic Pain Grade Scale (CPGS), Short Form-36 Bodily Pain Scale (SF-36 BPS), and Measure of Intermittent and Constant Osteoarthritis Pain (ICOAP). *Arthritis Care & Research*, *63 Suppl 11*, S240-252. https://doi.org/10.1002/acr.20543

Hewlett, S., Dures, E., & Almeida, C. (2011). Measures of fatigue: Bristol Rheumatoid Arthritis Fatigue Multi-Dimensional Questionnaire (BRAF MDQ), Bristol Rheumatoid Arthritis Fatigue Numerical Rating Scales (BRAF NRS) for severity, effect, and coping, Chalder Fatigue Questionnaire (CFQ), Checklist Individual Strength (CIS20R and CIS8R), Fatigue Severity Scale (FSS), Functional Assessment Chronic Illness Therapy (Fatigue) (FACIT-F), Multi-Dimensional Assessment of Fatigue (MAF), Multi-Dimensional Fatigue Inventory (MFI), Pediatric Quality Of Life (PedsQL) Multi-Dimensional Fatigue Scale, Profile of Fatigue (ProF), Short Form 36 Vitality Subscale (SF-36 VT), and Visual Analog Scales (VAS). *Arthritis Care & Research*, *63 Suppl 11*, S263-286. https://doi.org/10.1002/acr.20579

INSEE. (2012). *Population par âge − Tableaux de l’Économie Française | Insee*. https://www.insee.fr/fr/statistiques/1372600?sommaire=1372680

INSEE. (2017). *4 millions de seniors seraient en perte d’autonomie en 2050—Insee Première—1767*. https://www.insee.fr/fr/statistiques/4196949

Jeannerod. (2001). Neural Simulation of Action: A Unifying Mechanism for Motor Cognition. *NeuroImage*, *14*(1), S103–S109. https://doi.org/10.1006/nimg.2001.0832

Kharat, & Simonet. (2013). Outils diagnostiques de l’état confusionnel aigu. *Revue Médicale Suisse*. https://www.revmed.ch/RMS/2013/RMS-370/Outils-diagnostiques-de-l-etat-confusionnel-aigu

Kubicki, A., & Mourey, F. (2015). Rééducation gériatrique: Approche systémique. *EMC Kinésithérapie–Médecine Physique–Réadaptation*, *11*(4), 1–9.

Lundin-Olsson, L., Nyberg, L., & Gustafson, Y. (1997). “Stops walking when talking” as a predictor of falls in elderly people. *Lancet*, *349*(9052), 617. https://doi.org/10.1016/S0140-6736(97)24009-2

McKiernan, F. E. (2005). A simple gait-stabilizing device reduces outdoor falls and nonserious injurious falls in fall-prone older people during the winter. *Journal of the American Geriatrics Society*, *53*(6), 943–947. https://doi.org/10.1111/j.1532-5415.2005.53302.x

Menant, J. C., Schoene, D., Sarofim, M., & Lord, S. R. (2014). Single and dual task tests of gait speed are equivalent in the prediction of falls in older people: A systematic review and meta-analysis. *Ageing Research Reviews*, *16*, 83–104. https://doi.org/10.1016/j.arr.2014.06.001

Menant, J. C., Steele, J. R., Menz, H. B., Munro, B. J., & Lord, S. R. (2008). Optimizing footwear for older people at risk of falls. *Journal of Rehabilitation Research and Development*, *45*(8), 1167–1181.

Perera, S., Mody, S. H., Woodman, R. C., & Studenski, S. A. (2006). Meaningful change and responsiveness in common physical performance measures in older adults. *Journal of the American Geriatrics Society*, *54*(5), 743–749. https://doi.org/10.1111/j.1532-5415.2006.00701.x

Préfaut, & Ninot. (2009). *La Réhabilitation du malade respiratoire chronique*. https://www.elsevier-masson.fr/la-rehabilitation-du-malade-respiratoire-chronique-9782294048074.html

Professional Associations for Physical Activity, Sweden. (2010). *Physical Activity in the Prevention and Treatment of Disease—Folkhälsomyndigheten*. http://www.folkhalsomyndigheten.se/publicerat-material/publikationsarkiv/p/physical-activity-in-the-prevention-and-treatment-of-disease/

Rulleau, T., Mauvieux, B., & Toussaint, L. (2015). Influence of circadian rhythms on the temporal features of motor imagery for older adult inpatients. *Archives of Physical Medicine and Rehabilitation*, *96*(7), 1229–1234. https://doi.org/10.1016/j.apmr.2015.02.015

Seichi, A., Hoshino, Y., Doi, T., Akai, M., Tobimatsu, Y., Kita, K., & Iwaya, T. (2014). Determination of the optimal cutoff time to use when screening elderly people for locomotive syndrome using the one-leg standing test (with eyes open). *Journal of Orthopaedic Science*, *19*(4), 620–626. https://doi.org/10.1007/s00776-014-0581-8

Temfemo, A., & Ahmaidi, S. (2018). Variation d’utilisation d’aide technique à la marche par la personne âgée en EHPAD. *Neurophysiologie Clinique*, *48*(6), 333. https://doi.org/10.1016/j.neucli.2018.10.060

Vass, C., Edwards, C., Smith, A., Sahota, O., & Drummond, A. (2015). What do patients wear on their feet? A service evaluation of footwear in elderly patients. *International Journal of Therapy and Rehabilitation*, *22*(5), 225–232. https://doi.org/10.12968/ijtr.2015.22.5.225

# Liste des annexes

***Annexe 1 -***  Questionnaire Short-Falls Efficacy Scale International

***Annexe 2 –*** Confusion Assessment Method

***Annexe 3 –*** Notice des chaussettes antidérapantes *Medline*

## Annexe 1 : Short-Falls Efficacy Scale International

<http://www.profane.eu.org/documents/SHORT_FES-I/Short_FES-I_Swiss_French.pdf>

## Annexe 2 : Confusion Assessment

<https://www.revmed.ch/RMS/2013/RMS-370/Outils-diagnostiques-de-l-etat-confusionnel-aigu>

## Annexe 3 : Notice des Chaussettes antidérapantes medline
